# Distribution of the anther-smut pathogen *Microbotryum* on species of the Caryophyllaceae

**DOI:** 10.1111/j.1469-8137.2010.03268.x

**Published:** 2010-07

**Authors:** Michael E Hood, Jorge I Mena-Alí, Amanda K Gibson, Bengt Oxelman, Tatiana Giraud, Roxana Yockteng, Mary T K Arroyo, Fabio Conti, Amy B Pedersen, Pierre Gladieux, Janis Antonovics

**Affiliations:** 1 Department of Biology, Amherst CollegeAmherst, MA, USA; 2 Department of Plant and Environmental Sciences, University of GothenburgGothenburg, Sweden; 3 Ecologie, Systématique et Evolution, Centre National de la Recherche Scientifique, Université Paris-SudF-91405 Orsay Cedex, France; 4 MNHN, Département Systématique et Evolution16 rue Buffon CP 39, 75005 Paris, France; 5 Institute of Ecology and Biodiversity (IEB), Facultad de Ciencias, University of ChileCasilla 653, Santiago, Chile; 6 Dipartimento di Scienze Ambientali, UniversitÃ di Camerino-Centro Ricerche Floristiche dell’AppenninoBarisciano (L’Aquila), Italy; 7 Institute of Evolutionary Biology, University of EdinburghEdinburgh, UK; 8 Department of Biology, University of VirginiaCharlottesville, VA, USA

**Keywords:** anther smut, Caryophyllaceae, disease risk, herbarium, life history evolution, *Microbotryum*, *Silene*, *Ustilago violacea*

## Abstract

- Understanding disease distributions is of fundamental and applied importance, yet few studies benefit from integrating broad sampling with ecological and phylogenetic data. Here, anther-smut disease, caused by the fungus *Microbotryum*, was assessed using herbarium specimens of *Silene* and allied genera of the Caryophyllaceae.
- A total of 42 000 herbarium specimens were examined, and plant geographical distributions and morphological and life history characteristics were tested as correlates of disease occurrence. Phylogenetic comparative methods were used to determine the association between disease and plant life-span.
- Disease was found on 391 herbarium specimens from 114 species and all continents with native *Silene*. Anther smut occurred exclusively on perennial plants, consistent with the pathogen requiring living hosts to overwinter. The disease was estimated to occur in 80% of perennial species of *Silene* and allied genera. The correlation between plant life-span and disease was highly significant while controlling for the plant phylogeny, but the disease was not correlated with differences in floral morphology.
- Using resources available in natural history collections, this study illustrates how disease distribution can be determined, not by restriction to a clade of susceptible hosts or to a limited geographical region, but by association with host life-span, a trait that has undergone frequent evolutionary transitions.

## Introduction

Theoretical studies have emphasized that epidemiological and evolutionary dynamics may be heavily influenced by details of the pathogen’s and the host’s life cycles (Anderson & May, 1981; Thrall & Burdon, 2004; Barrett *et al.*, 2008). However, surprisingly few examples are available of diseases in natural populations where broad geographical sampling can be combined with ecological and phylogenetic data to understand pathogen distributions. Because endemic pathogens and their hosts are affected by long-term co-evolutionary processes, phylogenetic analyses can be combined with ecological and geographical data to identify the population, life history, and genetic traits that contribute to disease spread and persistence. Such an integrated approach can also be used to evaluate ecological factors and life history traits that put hosts at risk for the emergence of new diseases.

With the objective of assessing host characteristics that may influence the distribution of disease, we present here the results of a world-wide survey of the incidence of anther-smut disease using a survey of over 42 000 herbarium specimens of plant species in the genus *Silene* and allied genera of the Caryophyllaceae. The disease is vectored by insect pollinators and replaces the pollen in anthers of infected plants with fungal spores. The dark fungal spores in the anthers of infected plants can be recognized on herbarium specimens and confirmed by simple microscopy. However, the disease is sufficiently inconspicuous that plant collections appear to have been made without awareness of the disease (Antonovics *et al.*, 2003; Hood & Antonovics, 2003). Similar surveys of herbarium specimens have proven increasingly useful for understanding a variety of other naturally occurring diseases (Evans, 1987; Alexander *et al.*, 2007; Malmstrom *et al.*, 2007).

Anther-smut disease is caused by the basidiomycete fungus *Microbotryum violaceum sensu lato*. Although relatively uniform morphologically, *M. violaceum* on the Caryophyllaceae forms a large species complex with many specialized and evolutionarily independent lineages, some of which have been elevated recently to the level of distinct species (Lutz *et al.*, 2005; Le Gac *et al.*, 2007; Giraud *et al.*, 2008). Anther smut is one of the best studied nonagricultural diseases of plants, particularly in the context of pathogen ecology, phylogeny, and genetics (Antonovics *et al.*, 2002; Garber & Ruddat, 2002; Giraud *et al.*, 2008; Bernasconi *et al.*, 2009).

Anther-smut epidemiology can be driven by the host’s physiological resistance and by traits such as host life-span, mating system, floral displays, and pollinator specificity (Thrall *et al.*, 1993; Marr & Delph, 2005). Such fundamental plant traits are known to be under intense selection across diverse environmental and ecological conditions (Crawford & Abbott, 1994; Giles *et al.*, 2006), and they vary to a large degree among caryophyllaceous plants (Desfeux *et al.*, 1996; Kephart *et al.*, 2006). Combined with theoretical models on how this disease interacts with host life history traits, Thrall *et al.* (1993) showed that published reports of anther smut on the Caryophyllaceae were most frequent for perennial plant species and outcrossing subfamilies, with a trend toward species with larger flowers being more likely to be diseased. The survey by Thrall *et al.* (1993) included species from Europe and North America, which was the recognized range of the pathogen at the time. While the Caryophyllaceae has a world-wide distribution, it has been commonly accepted that anther-smut fungi are largely limited to temperate and subarctic regions of the Northern Hemisphere, although targeted surveys have not yet been conducted in other regions (but see Piepenbring, 2001; Marr & Delph, 2005). A tacit assumption has been that the pathogen’s geographical range is limited by climate, with cooler temperatures favoring transmission, or by latitudinal changes in the proportion of annual to perennial species.

To test the generality of the patterns described above, data on the presence of anther-smut disease in herbarium specimens were analyzed for associations with plant life-span, floral characteristics, and geographical distributions. This research greatly expands upon the previous study by Thrall *et al.* (1993), where the incidence of anther smut in relation to floral and life history characters was largely based on a literature survey, and geographical distributions and host phylogeny were not explicitly considered. Our goals were: first, to determine the degree to which the disease is confined to perennial plant species; secondly, to predict the proportion of perennial species expected to harbor the disease in nature; thirdly, to assess whether disease frequencies among perennials were associated with the plants’ geographical distributions or floral characteristics; and fourthly, to establish whether these relationships had evolved independently with regard to the host phylogeny. To establish whether anther-smut disease observed on specimens from distant hosts or geographical regions could be included in the discussion of fungi in the genus *Microbotryum*, we also carried out a limited phylogenetic study on a subset of pathogen samples from the herbaria using DNA sequencing. The current study covers a much larger geographical area and a more inclusive list of plant species than previous investigations (Thrall *et al.*, 1993; Antonovics *et al.*, 2003). The study reveals the nearly global distribution of anther-smut disease on the Caryophyllaceae while also assessing the relationships between infection and host traits in a quantitative and phylogenetic context.

## Materials and Methods

### Natural history

Anther-smut disease on the Caryophyllaceae is caused by fungal species of the genus *Microbotryum* (Basidiomycetes: Microbotryales). Related fungal pathogens, often classified in separate genera, are found on other plant families, including the Dipsacaceae, Lamiaceae, Polygonaceae, and Portulacaceae (Kemler *et al.*, 2006, 2009). Although sometimes referred to as a single species, the name *Microbotryum violaceum* represents a suite of host-specific species that remain incompletely resolved taxonomically (Le Gac *et al.*, 2007; Lutz *et al.*, 2008; Denchev *et al.*, 2009); therefore the genus name *Microbotryum* is used hereafter. Many species in the Caryophyllaceae are hosts to anther smut, particularly within the genus *Silene* (Thrall *et al.*, 1993). Anther-smut disease sterilizes infected hosts by causing female structures to abort and replacing pollen with powdery, dark-colored fungal spores. The disease is transmitted primarily by pollinators visiting infected flowers (Antonovics & Alexander, 1992; Biere & Honders, 1998).

The Caryophyllaceae consists almost exclusively of herbaceous plant species, representing a diverse range of ecologies and life histories from annuals to extremely long-lived perennials (e.g. Desfeux *et al.*, 1996; Forbis & Doak, 2004; Kephart *et al.*, 2006). Within this family, anther-smut disease is most common on the tribe *Sileneae* (Oxelman *et al.*, 2001; Table 2; Thrall *et al.*, 1993). Europe and Asia contain the largest numbers of species in the *Sileneae*, with smaller numbers found in Africa and North America and yet fewer in South America (Heywood, 1978). A suggested phylogeographical history of the genus *Silene* is a Eurasian origin followed by migration into the Americas via the Beringian region (Popp *et al.*, 2005; Popp & Oxelman, 2007). There are no native members of the *Sileneae* in Australia, although some species have become naturalized following introduction.

### Herbarium surveys

Specimens were examined from the following plant herbaria (with herbarium abbreviations according to Holmgren & Holmgren, 1998): European: Centro di Ricerche Floristiche dell’Appennino (APP); Botánico de Barcelona (BC); Instituto Museo di Storia Naturale dell’Università, Firenze (FI); Royal Botanical Gardens, Kew (K); Real Jardín Botánico de Madrid (MA); Muséum National d’Histoire Naturelle, Paris (P); North American: Gray Herbarium, Harvard University (GH); Missouri Botanical Garden (MO); New York Botanical Garden (NY); Smithsonian Institution, Washington, DC (US); African: Bolus Herbarium (BOL); South American: Museo Nacional de Historía Natural, Santiago, Chile (SGO); Asian: Jiangsu Institute of Botany, Nanjing (NAS). The survey focused on the genus *Silene* and other genera in the tribe *Sileneae*. Some members of the subfamilies Caryophylloideae (i.e. *Saponaria*, *Dianthus*, and *Petrorhagia*) and Alsinoideae (i.e. *Stellaria*) were also examined (Table 1), as were North and South American members of the genus *Calandrinia* in the Portulacaceae as a result of the recent discovery of an anther smut on *Calandrinia* that is related to *Microbotryum* on *Silene* (Le Gac *et al.*, 2007). Each herbarium sheet was examined for specimens with diseased flowers, and counts were based on total number of sheets with or without diseased specimens; no attempt was made to distinguish individual plants (see Hood & Antonovics, 2003). Locality information from herbarium labels of the diseased specimens was recorded and used to generate a distribution map of anther-smut disease found in this survey. In no case did we find any annotation indicating that the collector might have noticed that specimens on the sheets were diseased.

**Table 1:** Number of herbarium specimens examined for anther-smut disease classified by plant genus and life-span

Family Caryophyllaceae					
Subfamily Caryophylloideae					
		Total	Perennial	Annual	Undetermined
Tribe *Sileneae*	*Silene*	37 275 (728)	24 136 (514)	13 008 (141)	131 (73)
	*Lychnis*	1471 (31)	1469 (29)	1 (1)	1 (1)
	*Heliosperma*	275 (7)	275 (7)	0 (0)	0 (0)
	*Viscaria*	501 (3)	501 (3)	0 (0)	0 (0)
	*Atocion*	542 (4)	542 (4)	0 (0)	0 (0)
	*Eudianthe*	530 (2)	0 (0)	530 (2)	0 (0)
	*Agrostemma*	6 (1)	0 (0)	6 (1)	0 (0)
Remaining Caryophylloideae	*Saponaria*	336 (67)	282 (38)	8 (3)	46 (26)
	*Dianthus*	482 (24)	431 (8)	36 (8)	15 (8)
	*Petrorhagia*	21 (2)	11 (1)	10 (1)	0 (0)
Remaining Caryophyllaceae	*Stellaria*	398 (4)	398 (4)	0 (0)	0 (0)
Family Portulaceae	*Calandrinia*	772 (72)	311 (21)	425 (40)	36 (11)
	*Cistanthe*	3 (2)	3 (2)	0 (0)	0 (0)
	*Ceraria*	20 (4)	20 (4)	0 (0)	0 (0)
	*Calyptrotheca*	77 (3)	0 (0)	77 (3)	0 (0)
	Total	42 709 (955)	28 379 (636)	14 101 (200)	229 (119)

Numbers of species per genus are shown in parentheses. The tribe *Sileneae* is as defined in Oxelman *et al.* (2001).

**Table 2:** Number of diseased specimens found in herbaria, classified by plant genus and life-span

Family Caryophyllaceae					
Subfamily Caryophylloideae					
		Total	Perennial	Annual	Undetermined
Tribe *Sileneae*	*Silene*	316 (95)	316 (95)	0 (0)	0 (0)
	*Lychnis*	32 (7)	32 (7)	0 (0)	0 (0)
	*Heliosperma*	1 (1)	1 (1)	0 (0)	0 (0)
	*Viscaria*	10 (2)	10 (2)	0 (0)	0 (0)
	*Atocion*	3 (2)	3 (2)	0 (0)	0 (0)
	*Eudianthe*	0 (0)	0 (0)	0 (0)	0 (0)
	*Agrostemma*	0 (0)	0 (0)	0 (0)	0 (0)
Remaining Caryophylloideae	*Saponaria*	1 (1)	1 (1)	0 (0)	0 (0)
	*Dianthus*	9 (2)	9 (2)	0 (0)	0 (0)
	*Petrorhagia*	0 (0)	0 (0)	0 (0)	0 (0)
Remaining Caryophyllaceae	*Stellaria*	1 (1)	1 (1)	0 (0)	0 (0)
Family Portulaceae	*Calandrinia*	18 (3)	18 (3)	0 (0)	0 (0)
	*Cistanthe*	0 (0)	0 (0)	0 (0)	0 (0)
	*Ceraria*	0 (0)	0 (0)	0 (0)	0 (0)
	*Calyptrotheca*	0 (0)	0 (0)	0 (0)	0 (0)
	Total	391 (114)	391 (114)	0 (0)	0 (0)

Numbers of species with disease per genus are shown in parentheses.

To determine how representative the herbarium survey was of the species in the tribe *Sileneae*, a simulation analysis was performed based upon resampling specimens from the compiled data set. Entries were chosen from the data set of over 40 000 *Sileneae* specimens at random and with replacement, and each was assessed for whether the plant species had been sampled previously in the simulation. The expected number of new plant species added for each additional herbarium specimen was determined. After the first 20 000 specimens the rate of new plant species per specimen had reached a relatively constant value of *c*. 0.003. We therefore concluded that the database was sufficiently large and representative to provide a thorough survey of the Sileneae.

### Plant characteristics

To assess factors that may explain the disease frequencies, information was compiled for the life-span (annual or perennial), petal limb length, and flower color of each species (see Supporting Information Table S1). Species listed as biennial were included with annuals, and species listed variably as annual/perennial or biennial/perennial were included with perennials. Biennial species were included with annuals because, like annuals, individuals do not exhibit repeated flowering which is necessary for the persistence of this pollinator-transmitted disease over successive seasons (Thrall *et al.*, 1993); moreover, species with a winter-annual habit are sometimes described in the literature as biennial. The floral trait of petal limb length was used as a proxy for flower size; a previous study had suggested a positive relationship between flower size and disease incidence (Thrall *et al.*, 1993). The darkness of flower color was also investigated because we were concerned that it might bias collection of diseased plants by collectors, the disease being more conspicuous on white-colored flowers. The darkness of flower color was assessed on a scale from 1 to 4: 1, white; 2, white-yellow, white-green, or white-pink; 3, pink, violet, red-white, or lilac; 4, red, red-violet, dark red, or dark violet.

Data on plant traits were gathered from various sources including floras (Flora of North America,Rabeler & Hartman, 2005; Flora of China, Zhou *et al.*, 2001; Atlas Florae Europaeae, Jalas & Suominen, 1988; Flora iberica, online at http://www.bioscripts.net/flora/) and published articles (Desfeux *et al.*, 1996; Jürgens *et al.*, 1996, 2002a,b; Burleigh & Holtsford, 2003; Popp *et al.*, 2005; Jürgens, 2006; Popp & Oxelman, 2007). Particular efforts were made to confirm species identifications and avoid cases of taxonomic synonymy, principally using the *Sileneae* database (http://www.sileneae.info/boxweb), International Plant Names Index (http://www.ipni.org), and the Atlas Florae Europaeae (Jalas & Suominen, 1988). While systematic revisions are ongoing, this study used the current state of species identifications as operational taxonomic units for the analysis of disease distributions.

### Rates of disease

The frequency of disease within a species was assessed using the proportion of herbarium sheets with plants showing anther-smut symptoms.

To identify species that showed higher or lower disease frequencies than expected, while accounting for differences in the number of specimens examined, we used the probability of deviation from random expectations (i.e. binomial distribution probabilities) based on the diseased proportion of all perennial specimens. This analysis was restricted to perennial species because the disease is absent from annuals (see Results section). Perennial species likely to be disease-free in nature were identified as those where no disease was found despite a binomial distribution probability of < 0.05 for finding zero diseased specimens out of the number of specimens examined based on the diseased proportion of all perennial specimens and employing a Bonferroni correction for multiple independent tests. Plant characteristics of flower size and color were tested for correlations with positive or negative deviations from expected numbers of diseased specimens of perennial species using the nonparametric Spearman’s rank test in spss version 12 (SPSS Inc., Chicago, IL, USA).

The percentage of perennial species in the tribe *Sileneae* expected to be diseased in nature was estimated using two methods. First, the per cent diseased was determined among species where the binomial probability of not finding disease was < 0.05, < 0.10 and < 0.20 given the number of sheets examined and the expected overall frequency of disease in perennials (see Results section). Secondly, a simulation approach was used. For each species, a binomial distribution probability was calculated for finding at least one diseased specimen. In the simulation, a species was scored as being diseased if this calculated probability was greater than a uniform random value between zero and one. This simulation attempts to estimate the percentage of species that are diseased by including all perennial species surveyed rather than only the most intensively surveyed. The average probability of perennial species being diseased was then calculated across all species weighted by the number of specimens per species, and the simulation was averaged over 1000 iterations.

### Phylogenetic basis of disease occurrence

To investigate whether there was a phylogenetic basis to high and low rates of disease incidence, a phylogeny was constructed using chloroplast ribosomal protein *rps16* intron sequences of *Silene* available in GenBank National Centre for Biotechnology Information (NCBI) (http://www.ncbi.nlm.nih.gov/). Accession numbers are available in Table S2. The species were categorized into three groups: perennials with high disease (positive deviations from expected numbers of diseased specimens), perennials with low disease (negative deviations from expected numbers of diseased specimens), and annual species. DNA sequences were available for 14 perennial species with high disease, and this number of species was chosen from the categories of perennials with low disease, and from annual species. DNA sequences were aligned using ClustalW (http://www.ebi.ac.uk/clustalw/), and phylogenies were reconstructed with the mega 4.0 software (Kumar *et al.*, 2004) by maximum parsimony analysis using the CNI heuristic search option, 100 random additions of sequences, and 1000 bootstrap pseudoreplicates. Bayesian posterior probabilities were determined using MrBayes version 3.1, and the model of molecular evolution that best fitted the data was determined using jModeltest (Posada, 2008). Under the Akaike information criterion (AIC), the data were best fitted by the TPM1uf + G model which assumes equals rates for three types of substitutions (see jModeltest manual for details) with a gamma distribution (G) of site-specific rates. The general time reversible (GTR) model with a gamma distribution (G) of site-specific rates was used in the Bayesian analysis because it is the most similar model to TPM1uf + G available in MrBayes 3.1, and it was ranked 8th among the 88 models compared with the AIC. Priors of state frequencies were left at default settings and Markov chains were initiated from a random tree and run until the average standard deviation of split frequencies remained below 0.01, that is, 600 000 iterations; posterior probabilities were derived from 9000 post-burn-in trees.

To test whether disease status was correlated with life-span (annual vs perennial) while controlling for the plant phylogeny, a continuous Markov model in a maximum likelihood framework was used, as described by Pagel (1994) and implemented in the program Mesquite 2.6 (Maddison & Maddison, 2009) with 1000 simulations. Evidence of phylogenetic signal for the discrete characters of annual vs perennial life-span and presence or absence of disease were tested in Mesquite 2.6 by comparing the observed state transition steps against a simulated distribution of state transition steps in which the character states were shuffled randomly among taxa (Maddison & Slatkin, 1991). In this analysis, a smaller number of observed state transition steps than expected from the randomized distribution (based upon 95% confidence intervals) would indicate that the discrete character is determined significantly by phylogenetic history; the simulated distribution of state transition steps was based on 1000 iterations. These analyses were intended to assess the distribution of plant life-span and disease status rather than to provide systematic revisions to the group, which are underway elsewhere.

### Host and pathogen distributions

Because annual species were not found to be diseased (see Results section), we focused on the distributions of perennial species. Information on the geographical distributions of perennial *Silene* species in Europe was obtained from the Atlas Florae Europaeae Database (AFE) (http://www.fmnh.helsinki.fi/english/botany/afe/publishing/database.htm). These data were used to generate species richness maps. For comparison with the perennial species richness map, 10 European *Silene* species were chosen with the most significant binomial distribution probabilities for positive deviations from the expected number of diseased specimens, and 10 European *Silene* species were chosen with the largest numbers of examined specimens where no disease was found. This strategy was used to maximize confidence in assigning species to the two categories of plants with regard to observed rates of disease; the number of species to include in the heavily diseased category was arbitrarily set at a positive binomial distribution probability of 0.05, totaling 10 species in the AFE database, and an equal number of disease-free species was then also included. In addition, 10 annual *Silene* species were chosen with the largest numbers of examined specimens and used to generate a European distribution map. Among the European perennial species the correlation between the size of geographical ranges and the positive or negative deviation from expected numbers of diseased specimens was tested using the Spearman’s rank test in spss. The geographical range for each species was determined from the total number of 50 × 50 km grid points occupied by a species in the AFE database. Locality data recorded from herbarium labels of diseased specimens were used to generate a global distribution map of anther-smut disease found in the survey.

### Pathogen DNA sampling and analysis

The ability to obtain *Microbotryum* from herbarium samples raises the question of whether it is still possible to isolate DNA and characterize relationships among these samples, especially as some of these specimens would be difficult to resample in the wild. We therefore tested whether DNA could be obtained from herbarium collected smut. We included anther-smut samples infecting the host genus *Calandrinia*, because recent phylogenetic studies (Le Gac *et al.*, 2007) suggested that the anther smut from this genus (in the family Portulacaceae) falls within the clade containing anther smuts from the genus *Silene*. We therefore sought to confirm this in our sampling of anther smut from *Calandrinia* based on a larger number of herbarium specimens.

During the herbarium survey, samples of the infected anthers were collected, with permission, from some diseased specimens (see Fig. 4). DNA was extracted from infected anthers using the DNeasy Mini Kit (Qiagen), and PCR was performed using primers that anneal to the variable regions of the internal transcribed spacer region of the nuclear rRNA genes to amplify only *Microbotryum* DNA (intraITS forward: 5′-CTGTTTAACCAGGGCGTGAC; intraITS reverse: 5′-TGATCTCGAAGGTTAGGATGC). Accession numbers are available in Table S2. Field-collected material of *Microbotryum* from several hosts was also included as positive controls (see Fig. 4). DNA sequences were aligned using ClustalW, and phylogenies were reconstructed with the mega 4.0 software by maximum parsimony analysis using the CNI heuristic search option, 100 random additions of sequences, and 1000 bootstrap pseudoreplicates. Bayesian posterior probabilities for support of tree topology were determined as described in the section on Phylogenetic basis of disease occurrence.

## Results

### Rates of disease

The herbarium surveys included 42 707 specimens from 952 plant species, with the great majority belonging to tribe *Sileneae* and to other members of the Caryophylloideae subfamily (Table 1). Anther-smut disease was found on 391 herbarium specimens, which corresponded to disease on 114 plant species (Table 2). Anther-smut disease was present exclusively in perennial species and not in annuals (1.38% among 28 379 specimens of perennials, and no cases of disease among 14 101 specimens of annuals). Disease occurred on perennial species of all major taxonomic groups examined in this study (Table 2). The complete species list represents the most inclusive survey of this group to date (see Table S1 ).

The annual/perennial status of some species could not be determined from the literature (Table 1), but none of these was found to be diseased and most were represented by very few specimens (average = 1.9). The average number of specimens per species was 42 for perennials and 55 for annuals. Herbarium specimens labeled only with the genus name (e.g. ‘*Silene* indet.’) were examined but not included in further analyses; anther-smut disease was found on four of 178 *Silene* indet. specimens and four of 26 *Lychnis* indet. specimens.

In the tribe *Sileneae*, an estimated 84% of all perennial species are likely to be hosts to anther-smut disease in nature based on the data for species with a probability of not finding disease of < 0.05 (i.e. 26 out of 31 species with > 210 specimens having at least one that was diseased). Similar estimates were obtained by including *Sileneae* perennials with fewer herbarium specimens (i.e. where the probability of not finding disease if it were present was statistically less significant); the percentage of species found to be diseased was 82% for species with a *P*-value for not finding disease < 0.1 (*n* = 39 species, each with > 170 specimens), and 76% for a *P*-value < 0.2 (*n* = 54 species, each with > 120 specimens).

The simulation approach to estimate the proportion of perennial species with disease provided a comparable value of 81%. The percentage of examined *Sileneae* species that were classified as perennials vs annual was 79%. Therefore, with anther-smut disease on roughly 80% of perennials and an estimated 750–850 extant *Sileneae* species (e.g. Oxelman *et al.*, 2001; Eggens *et al.*, 2007), it is predicted that between 470 and 530 species might be diseased in nature for this tribe alone.

Among the perennial *Sileneae* species where fewer diseased specimens were found than expected, only *Silene stellata*, with no disease among 824 specimens, had a binomial distribution probability < 0.05 of having disease based on the overall average incidence of disease in perennials after correction for multiple independent tests (*P*-value corrected for 450 independent tests of perennials without disease = 0.008). Other species with large numbers of specimens but where no disease was found included *Silene fortunei* (411 specimens) and *Silene involucrata* (403 specimens), but for these to be significant would require fewer than 13 independent tests.

Species with statistically greater numbers of diseased specimens than expected based on binomial distribution probabilities included *Silene saxifraga* (32 diseased specimens among 491 specimens), *Lychnis fulgens* (13 among 116 specimens), and *Silene parryi* (21 among 373 specimens).

**Figure 1:**
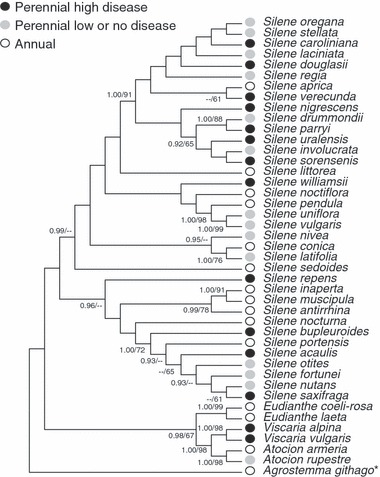
Phylogeny of plant species of the tribe *Sileneae* based upon maximum parsimony analysis of ribosomal protein *rps16* DNA sequences. Support values for tree topology are shown when they had values of Bayesian posterior probabilities/maximum parsimony bootstraps at least equal to 0.9/60, respectively. DNA sequences were obtained from GenBank National Centre for Biotechnology Information (NCBI) for an equal number of plant species in the categories of annuals (open circles), perennials with high disease frequencies (closed circles), and perennials with low or no disease (gray circles). Perennial species were chosen as those with the most significant binomial distribution probabilities for positive or negative deviations from overall disease frequencies among perennials. Annuals were chosen as those with the largest numbers of specimens examined. *Agrostemma githago was chosen as the outgroup to the remainder of the *Sileneae* based on Oxelman *et al.* (2001). Accession numbers are available in Table S2.

Analysis of the host phylogeny revealed that perennials with high disease frequencies were found together in multiple well-supported clades either with perennials having low or no disease, or with annual species (Fig. 1). There was no statistically significant evidence for a phylogenetic signal for either annual vs perennial life-span or presence or absence of disease; that is, the estimated number of state transition steps in the host phylogeny (*n* = 11) was not lower than expected by chance, indicating that these characters are highly labile during the evolution of the *Sileneae*. The association of life-span (annual vs perennial) and disease status was found to be statistically significant while controlling for the plant phylogeny (*P*-value from 1000 simulations = 0.001; independent log likelihood = 51.1985; correlated log likelihood = 43.5713).

No significant correlation of disease rates was found with flower size (correlation coefficient = −0.057, *P* = 0.476, *n* = 156) or with darkness of flower color (correlation coefficient = 0.083, *P* = 0.247, *n* = 195). Because these correlations were nonsignificant, they were not tested further by controlling for the plant phylogeny.

### Host and pathogen distributions

**Figure 2:**
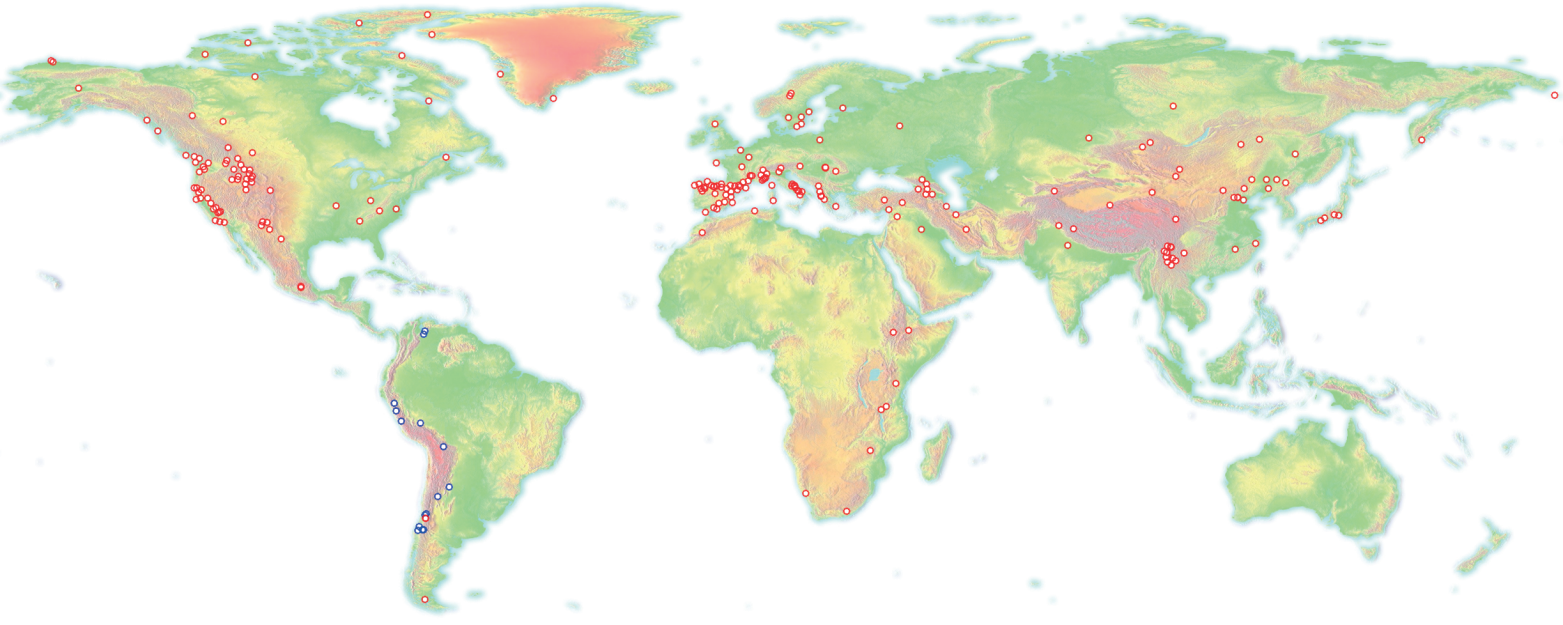
Distribution map of all diseased specimens found in this herbarium survey. Red markers, species in the Caryophyllaceae; blue markers, species of *Calandrinia* in the Portulacaceae. The map includes specimens with herbarium labels that were legible and contained locality data more specific than the country of origin. Continents are shaded according to land elevation.

The known geographical distribution of anther smuts (Fig. 2) was greatly expanded to include the presence of the pathogen in species of *Sileneae* from the Southern Hemisphere, both in South America (on *Silene chilensis* and *Silene magellanica*) and in southern regions of Africa (on *Silene burchellii*, *Silene ornata*, and *Silene undulata*). The difference between continents in the proportion of diseased perennial *Sileneae* specimens approached significance (χ^2^ = 8.41, df = 4, *P* = 0.078), and the trend was toward the least disease in the Southern Hemisphere and the most disease in Asia (Table 3).

**Table 3:** Frequency of disease among herbarium specimens of *Sileneae* from different continents

Continent	Specimens examined	Diseased specimens	Disease frequency
Africa	1521	12	0.0079
Asia	5375	89	0.0166
Europe	9123	125	0.0137
North America	8709	111	0.0127
South America	263	2	0.0076

**Figure 3:**
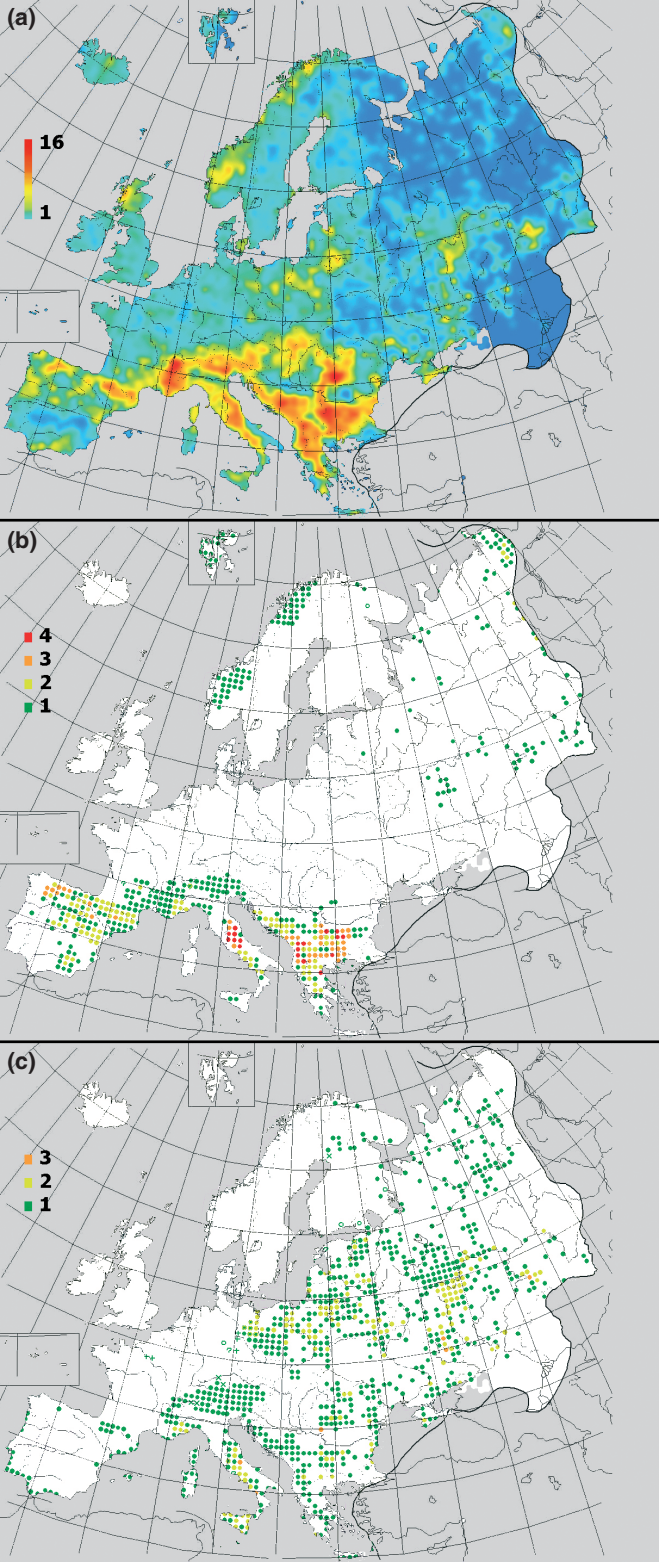
Species richness maps for perennial *Silene* species. (a) Distribution of all *Silene* species classified as perennial in the herbarium survey and included in the Atlas Florae Europaeae database (*n* = 104). (b) Distribution of the 10 most diseased *Silene* species, identified by the most significant positive deviations from expected number of diseased specimens based on binomial distribution probabilities. (c) Distribution of 10 *Silene* species with the largest number of examined species but where no disease was found. Color scales represent the numbers of overlapping species.

Within Europe, compiled distribution maps for perennial *Silene* species from the Atlas Florae Europaeae showed the highest species richness in southern mountain regions. Heavily diseased *Silene* species also appeared to be distributed in these regions (Fig. 3). By contrast, the most examined perennial species having no disease were more broadly distributed in regions of low *Silene* species richness. Annual *Silene* species exhibited a southern European distribution similar to that of perennial species, with each of the 10 annual species with the largest numbers of examined specimens overlapping in geographical distribution with the 10 most diseased perennial species (see Supporting Information Fig. S1). The geographical range size of perennial *Silene* in Europe was negatively correlated with the disease rates within species (Spearman’s rank correlation coefficient for all examined perennial species in the AFE database = −0.195, *P* = 0.048, *n* = 104; rank correlation coefficient including only diseased species = −0.456, *P* = 0.013, *n* = 29).

**Figure 4:**
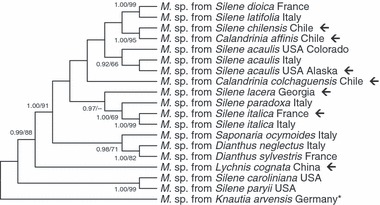
Phylogeny of *Microbotryum* (*M.*) based upon maximum parsimony analysis of DNA sequences for the internal transcribed spacer region of the nuclear rRNA genes. Support values for tree topology are shown when they had values of Bayesian posterior probabilities/maximum parsimony bootstraps at least equal to 0.9/60, respectively. The tree includes genotypes obtained from herbarium specimens (arrows) and from field collections that broadly represent the species diversity of *Microbotryum* species on the Caryophyllacaeae (see Le Gac *et al.*, 2007). *The outgroup pathogen on *Knautia arvensis* (Dipsacaceae) was chosen according to Kemler *et al.* (2006) and Lutz *et al.* (2008) and the sequence was obtained from GenBank National Centre for Biotechnology Information (NCBI). Accession numbers are available in Table S2.

Analysis of DNA sequence data from herbarium samples confirms that the lineage of *Microbotryum* causing anther smut on the Caryophyllaceae has representatives infecting other plant families (Le Gac *et al.*, 2007; but see Vánky, 1998, 2001). Samples collected from two species of South American *Calandrinia* (Fig. 2) group along with samples collected from species in the Caryophyllaceae rather than forming a sister clade (Fig. 4). Other species included in the phylogenetic analysis served as ‘controls’ and, as predicted, sequences of *Microbotryum* from herbarium specimens of *Silene italica* and *Silene acaulis* mapped adjacent to sequences of field-collected fungal specimens from the same host species. Moreover, sequences of anther smut on *S. chilensis* from South America fell within the clade containing *Microbotryum* from other *Silene* hosts.

## Discussion

Understanding the distribution of disease among related host species is a central challenge in pathogen ecology, especially in the context of disease emergence. Here we show that the hosts of anther-smut disease, caused by *Microbotryum* species, are exclusively perennial plants in the Caryophyllaceae. The occurrence of anther smut only on perennial plants is consistent with the disease lacking a free-living or environmentally resilient overwintering stage, but instead perennating inside the host and being spread directly from diseased flowers to healthy plants primarily by insect pollinators. This is in contrast to diverse fungal species in the Microbotryales (Kemler *et al.*, 2006) and Ustilaginales (Fischer & Holton, 1957) affecting tissues other than the anthers, such as floral smuts and seed smuts, where transmission appears to be primarily via spores overwintering on the seed coat or in the soil.

Using a search of the literature, Thrall *et al.* (1993) also found that the proportion of species with anther-smut disease was far greater in perennials than in annuals. However, in their study disease was reported in a substantial proportion of the annuals (*c*. 11% of the records of disease were on annual species). Unfortunately, the original data set from that study is no longer available, but the literature reports may have included specimens from botanical gardens or experimental inoculations. For example, Goldschmidt (1928) records having obtained *Silene noctiflora* from a botanical garden, and he successfully inoculated *Agrostemma githago* experimentally*.* We have also found that annual species can become diseased following artificial inoculation (M. E. Hood *et al*., unpublished results). Similarly, infection of annual species in the field may result from a transient cross-species transmission involving disease from sympatric perennials, but the maintenance of disease on an annual species alone is not expected to occur in nature.

Previous reports of seed-destroying smuts related to *Microbotryum* on several annual *Silene* species suggest that a change in the pathogen’s transmission ecology may be required to persist on hosts with contrasting life histories (e.g. annual vs perennial). Based largely on the location of spores and larger spore size, the seed-smut pathogen on annual *Silene* species (i.e. *Silene colorata*, *Silene crassipes* and *Silene apetala*; see Vánky, 2005) was recently removed from *Microbotryum* and renamed *Heradaea jehudana* (Denchev *et al.*, 2006). Development of smut spores in capsules, rather than anthers, suggests a nonvectored disease that is transmitted by environmental contamination, such that overlapping host generations are not required for disease persistence. Similar overwintering spores occur in *Microbotryum* species causing seed and ovary smuts on annuals from other plant families (Berner *et al.*, 2006; Denchev *et al.*, 2006). Whether the seed smut on annual *Silene* species is derived from an anther smut needs further study using molecular phylogenetics. However, Sloan *et al.* (2008) recently demonstrated that seed-smut disease symptoms can result simply from cross-species transmission of anther-smut-causing *Microbotryum* onto a novel *Silene* host. Moreover, the evolutionary transitions between anther and seed/ovary smuts appear to have occurred among *Microbotryum* lineages on the Caryophyllaceae (Kemler *et al.*, 2006). Thus, persistence of *Microbotryum* on hosts with alternate life histories (i.e. from perennials to annuals) may be facilitated by a concomitant and fundamental change in the pathogen’s ecology (i.e. from vector-borne to environmental transmission).

That the disease is restricted to perennial species raises the question of whether anther smut imposes selection in *Sileneae* in favor of an annual life history. Whether a species exhibits an annual or perennial habits appear to be highly labile in the *Sileneae*, with multiple evolutionary transitions and no statistically significant evidence of a phylogenetic signal. Anther smut is widely distributed within this group of plants and has been shown to constrain host population growth and persistence (Antonovics, 2004). However, Thrall *et al.* (1993) stressed that the selective role of diseases, which may be transient within populations, needs to be considered in comparison to other evolutionary forces affecting host life history traits. Moreover, the evolution of the annual habit is unlikely to be caused by anther-smut disease because an annual variant would gain little immediate advantage in terms of escaping disease in a population consisting largely of perennials. However, the interactions with anther smut may still have evolutionary consequences when, for example, the disease is lost from a species evolving a more annual habit or from regions where populations have an environmentally imposed annual habit. A genetic basis for physiological resistance to anther smut has been demonstrated in multiple *Silene* species (Alexander *et al.*, 1993; Cafuir *et al.*, 2007) and can be associated with fitness costs in the absence of disease (Biere & Antonovics, 1996). Therefore, the loss of anther smut could contribute to the success of annuals by favoring the abandonment of costly resistance mechanisms even if the disease was not a causal factor in the evolution of host life-span. These possibilities should encourage more detailed phylogenetic studies on this host–pathogen system so that the emergence of annual life histories can be assessed for ancestral associations with disease.

Overall, the probability that anther smut is present on a perennial species is remarkably high, particularly in the tribe *Sileneae*. The question then arises as to why some perennials are disease-free. No infected plants were found for *Silene stellata* despite a large number of specimens examined. This species is broadly distributed in eastern North America and is common across much of its range. We have not observed infected plants in the field, even in areas where its close relatives, *Silene caroliniana* and *Silene virginica*, are frequently diseased (see Antonovics *et al.*, 2003), and yet *S. stellata* can be readily infected by experimental inoculation (M. E. Hood *et al*., unpublished data). Other perennial species that so far appear healthy could also be targeted for more intensive herbarium surveys to establish disease-free status, which could then allow the assessment of shared traits that might discourage establishment of disease.

In the current study, we did not find any relationship between disease rates and flower size (as measured by petal limb length). While Thrall *et al.* (1993) initially speculated that larger flowers should attract larger pollinators that in turn should transmit disease at a higher rate and over larger distances, their results only approached significance at *P* < 0.1, and the analysis included both annual and perennial species. There was also not a significant correlation between disease rates and the darkness of flower color, which has previously been suggested as a potential confounding factor in how often disease is detected in the field or on herbarium specimens (Antonovics *et al.*, 2003).

Disease rate among perennials was negatively associated with the host’s geographical range size. There are at least two possible explanations for this result. First, species with large geographical ranges may occupy many habitats less suitable for *Microbotryum*. For example, the broadly distributed host *Silene vulgaris* is frequently diseased above 2000 m, but many herbarium specimens come from lower elevations where this species is largely disease-free (M. E. Hood *et al*., unpublished data). Disease on the host *Silene dioica* may follow a similar elevational gradient (Bucheli *et al.*, 2000). Secondly, very broadly distributed species may have much of their range in regions of low host species richness, and therefore experience a lower overall incidence of *Microbotryum* and fewer opportunities for host shifts, although the contribution of localized, transient host shifts to disease frequencies within host species is unknown. Both explanations are consistent with our observation that the distribution of species with low rates of disease appeared to overlap less with mountainous regions in southern Europe where we found the highest species richness of potential hosts. The current data are not sufficient to assess whether regions of high host diversity are ‘hotspots’ for disease emergence via host shifts, but this would be interesting to investigate in further studies. The importance of species richness has been suggested for emerging zoonotic diseases in humans (Jones *et al.*, 2008), and recent phylogenetic and experimental studies have shown the significance of host shifts in the Microbotryum system (Refrégier *et al.*, 2008).

The results demonstrate the broad distribution of anther-smut disease on the Caryophyllaceae. The full geographical distribution of anther-smut disease has not been widely recognized, particularly in the Southern Hemisphere. However, the herbarium survey was able to locate the pathogen on the Caryophyllaceae in the southernmost regions of South America (e.g. Punta Arenas, Chile) and Africa (e.g. near Port Elizabeth, South Africa). We have found one previous published report for the disease on *Silene* in South Africa, as a footnote (Reid & Hooper, 1957). The centers of diversity for *Sileneae* are in the Eastern Mediterranean and Central Asia (Eggens *et al.*, 2007), and our results provide suggestive evidence that anther smut is less frequent among perennial species whose distribution is the result of dispersal to the Americas or to the Southern Hemisphere. This raises the possibility either of a broader scale relationship between disease incidence and host species richness or that escape from the pathogen for ecological or genetic reasons was associated with ancient migration patterns of hosts into the Americas or southern continents (Popp *et al.*, 2005; Popp & Oxelman, 2007).

It should be emphasized that the data we present are not exhaustive for the distribution of anther smut. Many additional specific locations for the disease can be found in the literature, in mycological herbaria, and in previous field surveys of particular sets of host species or geographical regions (Antonovics *et al.*, 2003; Hood & Antonovics, 2003). The close relationship between the pathogen from South American *Calandrinia* (Portulacaceae) and *Silene* (Le Gac *et al.*, 2007) suggests the need to consider the distribution of *Microbotryum* outside the Caryophyllaceae. Moreover, the survey of specimens from nature does not fully reflect their actual geographical distributions because of a bias toward environments favored by botanists or toward countries where field studies have been more active. For example, the cluster of specimens in north-west Yunnan probably reflects the enormous collections by Père Jean Marie Delavay in the late 1800s and others who revisited that particular research area. Conclusions potentially influenced by unequal sampling effort should be confirmed by the collection of field data.

This study further demonstrates the utility of natural history collections for investigating large-scale disease distributions (Evans, 1987; Antonovics *et al.*, 2003; Alexander *et al.*, 2007; Malmstrom *et al.*, 2007). As with previous studies on *Microbotryum* (Antonovics *et al.*, 2003; Hood & Antonovics, 2003), there was little evidence from collectors’ notes or subsequent annotations that plant specimens were recognized as diseased. As further support that the data were not biased by the appearance of anther-smut symptoms, our analysis showed that disease rates were not correlated with either flower size or color, which could have affected how conspicuous the disease appears to collectors.
