# Supplementary material for: Distribution of the anther-smut pathogen Microbotryum on species of the Caryophyllaceae
Source: New Phytol. 2010 Jul;187(1):217–29. doi: 10.1111/j.1469-8137.2010.03268.x (PMC3487183; doi:10.1111/j.1469-8137.2010.03268.x)
Supplement: Supplementary file 1 [file nph0187-0217-SD1.doc]

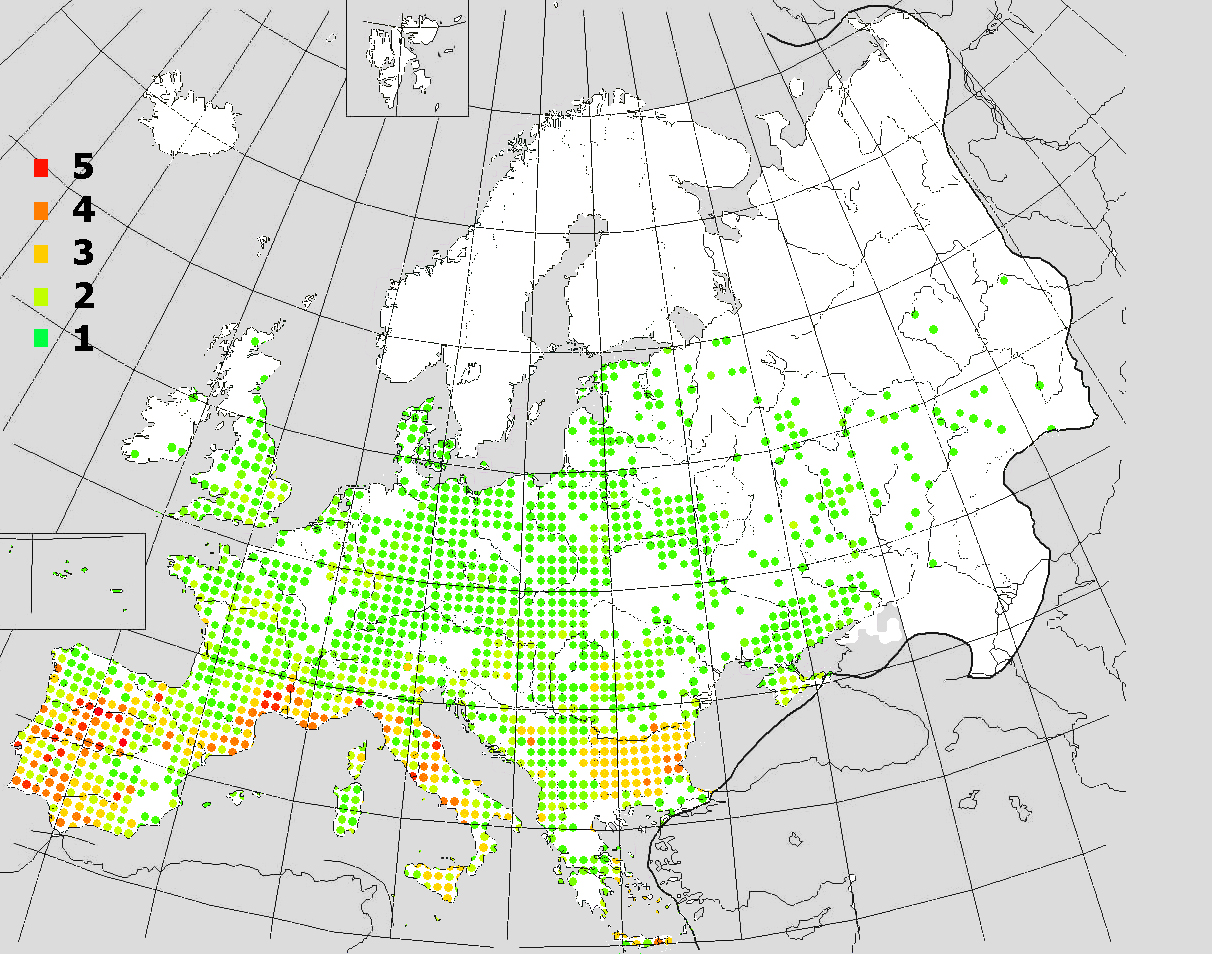


**Supporting Information Fig. S1** Species richness maps for annual *Silene* species including the ten annual species with the largest numbers of examined specimen. Information on the geographic distributions of annual *Silene* species in Europe was obtained from the Atlas Florae Europaeae Database (AFE) (www.fmnh.helsinki.fi/english/botany/afe/publishing/database.htm).
