# Supplementary material for: Distribution of the anther-smut pathogen Microbotryum on species of the Caryophyllaceae
Source: New Phytol. 2010 Jul;187(1):217–29. doi: 10.1111/j.1469-8137.2010.03268.x (PMC3487183; doi:10.1111/j.1469-8137.2010.03268.x)
Supplement: Supplementary file 2 [file nph0187-0217-SD2.doc]

**Supporting Information Table S1**

| **Species** | **Specimens Examined** | **Specimens Diseased** | **Lifespan** | **Color Scale (described in manuscript)** | **Petal Limb Length  (mm)** |
| --- | --- | --- | --- | --- | --- |
| *Silene gallica* | 1473 | 0 | Annual |  |  |
| *Silene vulgaris* | 1273 | 4 | Perennial | 1 | 8 |
| *Silene colorata* | 912 | 0 | Annual |  |  |
| *Silene nutans* | 887 | 6 | Perennial | 1 | 6 |
| *Silene stellata* | 824 | 0 | Perennial | 1 | 8 |
| *Silene menziesii* | 751 | 1 | Perennial | 1 | 3 |
| *Silene douglasii* | 745 | 14 | Perennial | 1 | 8 |
| *Silene laciniata* | 663 | 5 | Perennial | 4 | 11 |
| *Silene latifolia* | 649 | 7 | Perennial | 1 | 12 |
| *Silene antirrhina* | 639 | 0 | Annual |  |  |
| *Silene dichotoma* | 597 | 0 | Annual |  |  |
| *Silene burchellii* | 590 | 5 | Perennial | 3 | 8 |
| *Silene aprica* | 535 | 0 | Annual |  |  |
| *Silene uralensis* | 518 | 15 | Perennial | 4 | 2 |
| *Silene conoidea* | 516 | 0 | Annual |  |  |
| *Silene italica* | 506 | 4 | Perennial | 1 | 7 |
| *Silene dioica* | 499 | 1 | Perennial | 4 | 11 |
| *Silene saxifraga* | 491 | 32 | Perennial | 1 | 6 |
| *Silene nocturna* | 487 | 0 | Annual |  |  |
| *Silene conica* | 470 | 0 | Annual |  |  |
| *Lychnis flos-cuculi* | 434 | 6 | Perennial | 3 | 17 |
| *Silene repens* | 431 | 15 | Perennial | 2 | 6 |
| *Silene fortunei* | 411 | 0 | Perennial | 4 | 15 |
| *Atocion rupestre* | 408 | 1 | Perennial | 1 | 4 |
| *Silene involucrata* | 403 | 0 | Perennial | 1 | 6 |
| *Silene ciliata* | 401 | 14 | Perennial | 1 | 4 |
| *Silene otites* | 385 | 3 | Perennial | 2 | 3 |
| *Viscaria alpina* | 381 | 8 | Perennial | 3 | 5 |
| *Silene parryi* | 373 | 21 | Perennial | 1 | 6 |
| *Silene drummondii* | 369 | 1 | Perennial | 1 |  |
| *Silene noctiflora* | 358 | 0 | Annual |  |  |
| *Silene uniflora* | 349 | 2 | Perennial | 1 | 7 |
| *Silene scouleri* | 318 | 3 | Perennial | 2 | 6 |
| *Silene portensis* | 317 | 0 | Annual |  |  |
| *Eudianthe coeli-rosa* | 289 | 0 | Annual |  |  |
| *Silene muscipula* | 289 | 0 | Annual |  |  |
| *Silene scabriflora* | 289 | 0 | Annual |  |  |
| *Silene apetala* | 285 | 0 | Annual |  |  |
| *Silene boryi* | 285 | 2 | Perennial | 4 | 9 |
| *Atocion armeria* | 284 | 0 | Annual |  |  |
| *Silene tenuis* | 277 | 4 | Perennial |  |  |
| *Silene californica* | 260 | 1 | Perennial | 4 | 11 |
| *Silene acaulis* | 259 | 5 | Perennial | 3 | 4 |
| *Silene nicaeensis* | 254 | 0 | Perennial | 3 | 6 |
| *Lychnis coronaria* | 252 | 0 | Perennial | 4 | 15 |
| *Silene aegyptiaca* | 249 | 0 | Annual |  |  |
| *Silene diversifolia* | 245 | 0 | Annual |  |  |
| *Eudianthe laeta* | 241 | 0 | Annual |  |  |
| *Silene mellifera* | 223 | 1 | Perennial | 1 | 7 |
| *Silene inaperta* | 222 | 0 | Annual |  |  |
| *Calandrinia ciliata* | 216 | 0 | Annual |  |  |
| *Stellaria borealis* | 211 | 1 | Perennial |  |  |
| *Silene verecunda* | 205 | 5 | Perennial | 3 | 5 |
| *Silene viscosa* | 204 | 1 | Perennial | 1 | 20 |
| *Lychnis senno* | 201 | 2 | Perennial | 4 | 23 |
| *Silene lemmonii* | 201 | 2 | Perennial | 1 | 6 |
| *Silene legionensis* | 191 | 7 | Perennial | 2 | 7 |
| *Silene oregana* | 183 | 0 | Perennial | 1 | 6 |
| *Silene littorea* | 181 | 0 | Annual |  |  |
| *Silene nivea* | 176 | 0 | Perennial | 1 | 7 |
| *Silene behen* | 175 | 0 | Annual |  |  |
| *Silene regia* | 167 | 0 | Perennial | 4 | 20 |
| *Silene secundiflora* | 166 | 0 | Annual |  |  |
| *Silene sedoides* | 165 | 0 | Annual |  |  |
| *Silene tridentata* | 165 | 0 | Annual |  |  |
| *Silene villosa* | 163 | 0 | Annual |  |  |
| *Silene psammitis* | 157 | 0 | Annual |  |  |
| *Silene caroliniana* | 152 | 3 | Perennial | 2 | 11 |
| *Heliosperma alpestre* | 151 | 1 | Perennial | 1 |  |
| *Silene cserei* | 148 | 0 | Perennial | 1 | 7 |
| *Silene pendula* | 147 | 0 | Annual |  |  |
| *Silene capensis* | 146 | 1 | Perennial | 1 | 10 |
| *Silene spergulifolia* | 141 | 2 | Perennial |  |  |
| *Silene bellidifolia* | 140 | 0 | Annual |  |  |
| *Silene fuscata* | 138 | 0 | Annual |  |  |
| *Silene gonosperma* | 137 | 0 | Perennial | 3 | 3 |
| *Silene ramosissima* | 137 | 0 | Annual |  |  |
| *Silene campanulata* | 133 | 0 | Perennial | 1 | 7 |
| *Calandrinia acaulis* | 131 | 12 | Perennial |  |  |
| *Silene bernardina* | 129 | 1 | Perennial | 1 | 5 |
| *Silene firma* | 129 | 0 | Annual |  |  |
| *Silene sorensenis* | 128 | 2 | Perennial | 1 | 4 |
| *Silene paradoxa* | 126 | 1 | Perennial | 1 |  |
| *Silene fruticosa* | 125 | 0 | Perennial | 3 |  |
| *Silene undulata* | 124 | 3 | Perennial | 1 | 10 |
| *Stellaria graminea* | 121 | 0 | Perennial |  |  |
| *Atocion compactum* | 118 | 2 | Perennial | 2 |  |
| *Silene sericea* | 117 | 0 | Annual |  |  |
| *Silene supina* | 117 | 0 | Perennial | 1 | 12 |
| *Lychnis fulgens* | 116 | 13 | Perennial | 4 | 23 |
| *Silene sclerocarpa* | 116 | 0 | Annual |  |  |
| *Silene bupleuroides* | 114 | 3 | Perennial | 1 | 12 |
| *Silene nemoralis* | 114 | 1 | Perennial | 1 | 10 |
| *Saponaria officinalis* | 113 | 0 | Perennial |  |  |
| *Silene tatarinowii* | 112 | 2 | Perennial | 1 | 7 |
| *Silene vallesia* | 112 | 1 | Perennial | 1 | 11 |
| *Viscaria vulgaris* | 112 | 2 | Perennial | 3 | 7 |
| *Silene andryalifolia* | 111 | 2 | Perennial | 1 | 8 |
| *Silene coniflora* | 109 | 0 | Annual |  |  |
| *Silene hookeri* | 107 | 0 | Perennial | 2 | 18 |
| *Heliosperma pusillum* | 105 | 0 | Perennial | 2 | 5 |
| *Silene arabica* | 103 | 0 | Annual |  |  |
| *Silene cordifolia* | 99 | 0 | Perennial | 1 |  |
| *Silene graeca* | 99 | 0 | Annual |  |  |
| *Lychnis coronata* | 98 | 1 | Perennial | 3 | 23 |
| *Silene longipetala* | 97 | 0 | Perennial | 1 |  |
| *Silene chlorifolia* | 93 | 4 | Perennial |  | 13 |
| *Silene jenisseensis* | 92 | 1 | Perennial | 2 |  |
| *Silene odontopetala* | 92 | 3 | Perennial |  |  |
| *Saponaria pumilio* | 91 | 8 | Perennial |  |  |
| *Silene rotundifolia* | 89 | 0 | Perennial | 4 | 13 |
| *Silene hirsuta* | 88 | 0 | Annual |  |  |
| *Saponaria lutea* | 87 | 0 | Perennial |  |  |
| *Silene clandestina* | 87 | 0 | Annual |  |  |
| *Silene echinospermoides* | 87 | 0 | Annual |  |  |
| *Silene multicaulis* | 87 | 6 | Perennial | 2 | 8 |
| *Silene swertiaefolia* | 87 | 1 | Perennial |  |  |
| *Silene chlorantha* | 85 | 0 | Perennial |  |  |
| *Silene arenosa* | 84 | 0 | Annual |  |  |
| *Lychnis chalcedonica* | 83 | 0 | Perennial | 4 | 8 |
| *Lychnis flos-jovis* | 83 | 0 | Perennial | 3 |  |
| *Silene microsperma* | 82 | 0 | Annual |  |  |
| *Silene nuda* | 82 | 0 | Perennial | 3 | 8 |
| *Silene sargentii* | 82 | 5 | Perennial | 1 | 9 |
| *Silene tatarica* | 81 | 0 | Perennial | 1 |  |
| *Silene cappadocica* | 80 | 1 | Perennial | 2 |  |
| *Silene moorcroftiana* | 79 | 0 | Perennial | 3 |  |
| *Silene squamigera* | 79 | 0 | Annual |  |  |
| *Silene thorei* | 77 | 0 | Perennial | 1 |  |
| *Silene catholica* | 76 | 0 | Perennial | 1 |  |
| *Silene pseudoatocion* | 76 | 0 | Annual |  |  |
| *Silene obtusifolia* | 75 | 0 | Annual |  |  |
| *Silene cretica* | 73 | 0 | Annual |  |  |
| *Silene multiflora* | 73 | 3 | Perennial | 1 | 9 |
| *Silene asclepiadea* | 72 | 3 | Perennial | 3 | 5 |
| *Silene macrosolen* | 71 | 1 | Perennial |  |  |
| *Silene williamsii* | 71 | 2 | Perennial | 1 | 9 |
| *Silene linearis* | 68 | 0 | Annual |  |  |
| *Silene nigrescens* | 68 | 1 | Perennial | 4 | 5 |
| *Silene scaposa* | 68 | 0 | Perennial | 2 | 6 |
| *Silene succulenta* | 68 | 0 | Perennial | 1 |  |
| *Silene wolgensis* | 68 | 0 | Annual |  |  |
| *Silene viridiflora* | 67 | 0 | Perennial | 2 | 5 |
| *Silene micropetala* | 65 | 0 | Annual |  |  |
| *Silene magellanica* | 64 | 1 | Perennial | 2 |  |
| *Silene ampullata* | 63 | 0 | Perennial |  |  |
| *Silene glauca* | 63 | 0 | Annual |  |  |
| *Silene foetida* | 62 | 0 | Perennial | 3 | 8 |
| *Silene macrodonta* | 62 | 0 | Annual |  |  |
| *Silene corrugata* | 60 | 1 | Perennial |  |  |
| *Silene corsica* | 60 | 0 | Perennial |  |  |
| *Silene elisabethae* | 60 | 1 | Perennial | 3 |  |
| *Silene grayi* | 59 | 9 | Perennial | 3 | 4 |
| *Silene suksdorfii* | 59 | 4 | Perennial | 1 | 5 |
| *Silene delavayi* | 58 | 1 | Perennial | 4 | 10 |
| *Silene nodulosa* | 58 | 1 | Perennial | 2 |  |
| *Saponaria bellidifolia* | 57 | 0 | Perennial |  |  |
| *Silene crassipes* | 56 | 0 | Annual |  |  |
| *Silene aellenii* | 55 | 0 | Annual |  |  |
| *Silene bridgesii* | 55 | 0 | Perennial | 1 | 10 |
| *Silene edgeworthii* | 54 | 0 | Perennial |  |  |
| *Silene fruticulosa* | 54 | 0 | Perennial |  |  |
| *Silene ovata* | 54 | 0 | Perennial | 1 | 8 |
| *Silene vivianii* | 54 | 0 | Annual |  |  |
| *Silene velutina* | 53 | 0 | Perennial |  |  |
| *Silene petersonii* | 52 | 0 | Perennial | 3 | 15 |
| *Silene napuligera* | 50 | 1 | Perennial | 4 |  |
| *Silene racemosa* | 50 | 0 | Annual |  |  |
| *Lychnis miqueliana* | 49 | 5 | Perennial | 3 | 23 |
| *Silene chilensis* | 49 | 1 | Perennial | 1 |  |
| *Silene thysanodes* | 49 | 0 | Perennial |  |  |
| *Silene genovevae* | 48 | 0 | Perennial |  |  |
| *Silene multifida* | 46 | 1 | Perennial | 1 |  |
| *Silene chaetodonta* | 45 | 0 | Annual |  |  |
| *Silene occidentalis* | 45 | 0 | Perennial | 3 | 15 |
| *Silene procumbens* | 45 | 0 | Perennial |  |  |
| *Saponaria caespitosa* | 44 | 0 | Perennial |  |  |
| *Silene borderi* | 44 | 0 | Perennial | 2 | 6 |
| *Silene lanuginosa* | 44 | 0 | Perennial |  |  |
| *Silene papillosa* | 44 | 0 | Annual |  |  |
| *Silene pauciflora* | 44 | 0 | Perennial |  |  |
| *Silene viscidula* | 44 | 1 | Perennial | 4 | 5 |
| *Silene altaica* | 43 | 0 | Perennial | 1 |  |
| *Silene foliosa* | 43 | 0 | Annual |  |  |
| *Silene songarica* | 43 | 0 | Perennial | 3 |  |
| *Silene gracilis* | 42 | 0 | Annual |  |  |
| *Silene germana* | 41 | 0 | Annual |  |  |
| *Silene marschallii* | 41 | 0 | Perennial |  |  |
| *Silene polypetala* | 40 | 0 | Perennial | 2 | 20 |
| *Cistanthe monospermum* | 39 | 0 | Annual |  |  |
| *Dianthus barbatus* | 39 | 0 | Perennial |  |  |
| *Silene acutifolia* | 39 | 0 | Perennial | 3 | 8 |
| *Lychnis cognata* | 38 | 4 | Perennial | 4 | 23 |
| *Silene compacta* | 38 | 2 | Perennial | 3 | 9 |
| *Silene olympica* | 38 | 0 | Perennial |  |  |
| *Silene parishii* | 38 | 0 | Perennial | 2 | 8 |
| *Silene platyphylla* | 38 | 0 | Perennial | 3 | 5 |
| *Silene sendtneri* | 38 | 0 | Perennial |  |  |
| *Silene linnaeana* | 37 | 0 | Perennial | 1 | 4 |
| *Silene discolor* | 36 | 0 | Annual |  |  |
| *Silene indica* | 36 | 1 | Perennial | 3 | 4 |
| *Silene saxatilis* | 36 | 1 | Perennial |  |  |
| *Silene laevigata* | 35 | 0 | Perennial |  |  |
| *Stellaria aquatica* | 35 | 0 | Perennial |  |  |
| *Calandrinia compressa* | 34 | 0 | Annual |  |  |
| *Cistanthe monandrum* | 34 | 0 | Annual |  |  |
| *Saponaria ocymoides* | 34 | 1 | Perennial |  |  |
| *Silene aperta* | 34 | 4 | Perennial | 1 | 8 |
| *Silene aucheriana* | 34 | 2 | Perennial |  |  |
| *Silene multinervia* | 34 | 0 | Annual |  |  |
| *Silene otodonta* | 34 | 1 | Perennial | 3 | 7 |
| *Silene patula* | 34 | 0 | Perennial |  |  |
| *Silene stricta* | 34 | 0 | Annual |  |  |
| *Silene armena* | 33 | 0 | Perennial |  |  |
| *Silene caryophylloides* | 33 | 0 | Perennial |  |  |
| *Silene parviflora* | 33 | 3 | Perennial | 2 | 3 |
| *Silene sibirica* | 33 | 0 | Perennial | 2 | 9 |
| *Calandrinia affinis* | 32 | 2 | Perennial |  |  |
| *Silene radicosa* | 32 | 0 | Perennial |  |  |
| *Silene virginica* | 32 | 0 | Perennial | 4 | 23 |
| *Silene damascena* | 31 | 0 | Annual |  |  |
| *Silene gigantea* | 31 | 0 | Annual |  |  |
| *Silene ruprechtii* | 31 | 0 | Perennial |  |  |
| *Stellaria alsine* | 31 | 0 | Perennial |  |  |
| *Calandrinia maritima* | 30 | 0 | Annual |  |  |
| *Silene atocioides* | 30 | 0 | Annual |  |  |
| *Silene brahuica* | 30 | 0 | Perennial |  |  |
| *Silene disticha* | 30 | 0 | Annual |  |  |
| *Silene laxantha* | 30 | 0 | Perennial |  |  |
| *Silene melanantha* | 30 | 3 | Perennial | 3 | 5 |
| *Silene densiflora* | 29 | 0 | Perennial |  |  |
| *Silene dianthoides* | 29 | 0 | Perennial |  |  |
| *Silene diclinis* | 29 | 0 | Perennial | 2 | 8 |
| *Silene imbricata* | 29 | 0 | Annual |  |  |
| *Silene montbretiana* | 29 | 0 | Perennial |  |  |
| *Silene roemeri* | 29 | 2 | Perennial | 1 | 5 |
| *Silene stenophylla* | 28 | 1 | Perennial | 1 |  |
| *Silene argillosa* | 27 | 0 | Annual |  |  |
| *Silene mandonii* | 27 | 0 | Perennial |  |  |
| *Silene waldsteinii* | 27 | 5 | Perennial | 1 | 8 |
| *Silene caespitella* | 26 | 0 | Perennial |  |  |
| *Silene davidii* | 26 | 0 | Perennial | 4 | 7 |
| *Silene echinosperma* | 26 | 0 | Annual |  |  |
| *Silene gracilicaulis* | 26 | 0 | Perennial | 3 |  |
| *Silene nana* | 26 | 0 | Annual |  |  |
| *Silene spinescens* | 26 | 0 | Perennial |  |  |
| *Dianthus sylvestris* | 25 | 0 | Perennial |  |  |
| *Silene biappendiculata* | 25 | 0 | Perennial |  |  |
| *Silene crassifolia* | 25 | 0 | Perennial |  |  |
| *Silene linicola* | 25 | 0 | Annual |  |  |
| *Silene struthioloides* | 25 | 0 | Perennial |  |  |
| *Silene zawadskii* | 25 | 0 | Perennial |  |  |
| *Lychnis affinis* | 24 | 0 | Perennial |  |  |
| *Silene cashmeriana* | 24 | 0 | Perennial | 2 | 7 |
| *Silene cuatrecasasii* | 24 | 0 | Annual |  |  |
| *Silene integripetala* | 24 | 0 | Annual |  |  |
| *Silene ornata* | 24 | 1 | Perennial | 4 | 12 |
| *Silene reuteriana* | 24 | 0 | Annual |  |  |
| *Silene scopulorum* | 24 | 4 | Perennial | 3 | 4 |
| *Silene subconica* | 24 | 0 | Annual |  |  |
| *Calandrinia compacta* | 23 | 0 | Perennial |  |  |
| *Silene borysthenica* | 23 | 0 | Annual |  |  |
| *Silene campanula* | 23 | 5 | Perennial | 1 | 6 |
| *Silene nepalensis* | 23 | 0 | Perennial | 3 | 2 |
| *Silene primuliflora* | 23 | 0 | Perennial |  |  |
| *Silene thurberi* | 23 | 0 | Perennial | 2 | 3 |
| *Silene almolae* | 22 | 0 | Annual |  |  |
| *Silene arguta* | 22 | 0 | Perennial |  |  |
| *Silene commelinifolia* | 22 | 0 | Perennial |  |  |
| *Silene coutinhoi* | 22 | 0 | Perennial | 2 | 9 |
| *Silene gemmata* | 22 | 0 | Annual |  |  |
| *Silene graminifolia* | 22 | 0 | Perennial | 1 |  |
| *Silene libanotica* | 22 | 2 | Perennial | 1 | 7 |
| *Silene lydia* | 22 | 0 | Annual |  |  |
| *Silene lynesii* | 22 | 0 | Perennial |  |  |
| *Silene makmeliana* | 22 | 0 | Perennial |  |  |
| *Silene meyeri* | 22 | 0 | Perennial |  |  |
| *Lychnis divaricata* | 21 | 0 | Perennial |  |  |
| *Silene quadrifolia* | 21 | 0 | Unclassified |  |  |
| *Silene volubilitana* | 21 | 0 | Annual |  |  |
| *Calandrinia caespitosa* | 20 | 0 | Perennial |  |  |
| *Calandrinia colchaguensis* | 20 | 4 | Perennial |  |  |
| *Calandrinia grandiflora* | 20 | 0 | Perennial |  |  |
| *Silene corinthiaca* | 20 | 0 | Annual |  |  |
| *Silene eriocalycina* | 20 | 0 | Perennial |  |  |
| *Silene falcata* | 20 | 0 | Perennial |  |  |
| *Silene ibosii* | 20 | 0 | Annual |  |  |
| *Silene odoratissima* | 20 | 0 | Annual |  |  |
| *Silene rosulata* | 20 | 0 | Perennial |  |  |
| *Silene auriculifolia* | 19 | 0 | Perennial | 3 | 7 |
| *Silene plutonica* | 19 | 0 | Perennial |  |  |
| *Silene pruinosa* | 19 | 0 | Perennial |  |  |
| *Silene subciliata* | 19 | 0 | Perennial | 4 | 27 |
| *Silene yunnanensis* | 19 | 2 | Perennial | 3 | 8 |
| *Dianthus mooiensis* | 18 | 0 | Perennial |  |  |
| *Silene cardiopetala* | 18 | 1 | Perennial | 4 | 6 |
| *Silene grandiflora* | 18 | 0 | Perennial | 4 |  |
| *Silene himalayensis* | 18 | 0 | Perennial | 4 |  |
| *Silene hitchguirei* | 18 | 0 | Perennial | 2 | 4 |
| *Silene stenobotrys* | 18 | 0 | Perennial |  |  |
| *Lychnis grandiflora* | 17 | 0 | Perennial |  |  |
| *Silene cariensis* | 17 | 0 | Annual |  |  |
| *Silene marizii* | 17 | 0 | Perennial | 1 | 8 |
| *Silene peduncularis* | 17 | 0 | Perennial |  |  |
| *Silene rhynchocarpa* | 17 | 0 | Perennial |  |  |
| *Silene schafta* | 17 | 0 | Perennial |  |  |
| *Silene seelyi* | 17 | 0 | Perennial | 4 | 3 |
| *Silene spaldingii* | 17 | 0 | Perennial | 2 | 3 |
| *Silene ungeri* | 17 | 0 | Annual |  |  |
| *Dianthus basuticus* | 16 | 0 | Perennial |  |  |
| *Dianthus namaensis* | 16 | 0 | Perennial |  |  |
| *Dianthus zeyheri* | 16 | 0 | Perennial |  |  |
| *Heliosperma macranthum* | 16 | 0 | Perennial |  |  |
| *Silene bungei* | 16 | 2 | Perennial | 4 |  |
| *Silene kingii* | 16 | 0 | Perennial | 3 | 5 |
| *Silene kunawarensis* | 16 | 0 | Perennial |  |  |
| *Silene lagunensis* | 16 | 0 | Perennial |  |  |
| *Silene stewartii* | 16 | 0 | Perennial |  |  |
| *Atocion lerchenfeldianum* | 15 | 0 | Perennial |  |  |
| *Calandrinia diffusa* | 15 | 0 | Perennial |  |  |
| *Calandrinia monandra* | 15 | 0 | Annual |  |  |
| *Silene cambessedesii* | 15 | 0 | Annual |  |  |
| *Silene congesta* | 15 | 0 | Perennial |  |  |
| *Silene delicatula* | 15 | 0 | Annual |  |  |
| *Silene falconeriana* | 15 | 0 | Perennial |  |  |
| *Silene incurvifolia* | 15 | 0 | Perennial | 1 |  |
| *Silene juvenica* | 15 | 0 | Unclassified |  |  |
| *Silene lagenocalyx* | 15 | 0 | Annual |  |  |
| *Silene longicilia* | 15 | 0 | Perennial | 2 | 8 |
| *Silene quadrifida* | 15 | 0 | Perennial |  |  |
| *Calandrinia paniculata* | 14 | 0 | Perennial |  |  |
| *Dianthus monspessulanus* | 14 | 1 | Perennial |  |  |
| *Silene biafrae* | 14 | 0 | Perennial |  |  |
| *Silene chodatii* | 14 | 1 | Perennial | 3 | 8 |
| *Silene heterodonta* | 14 | 0 | Perennial |  |  |
| *Silene macrostyla* | 14 | 0 | Perennial | 1 | 6 |
| *Silene sartorii* | 14 | 0 | Annual |  |  |
| *Silene suaveolens* | 14 | 3 | Perennial | 1 | 18 |
| *Calandrinia salsoloides* | 13 | 0 | Annual |  |  |
| *Silene delphica* | 13 | 0 | Perennial |  |  |
| *Silene fabaria* | 13 | 0 | Perennial |  |  |
| *Silene flammulifolia* | 13 | 0 | Perennial |  |  |
| *Silene flavescens* | 13 | 0 | Perennial |  |  |
| *Silene gallinyi* | 13 | 0 | Annual |  |  |
| *Silene persica* | 13 | 1 | Perennial | 2 |  |
| *Silene primulifolia* | 13 | 0 | Perennial |  |  |
| *Silene reticulata* | 13 | 0 | Perennial |  |  |
| *Calandrinia alba* | 12 | 0 | Unclassified |  |  |
| *Dianthus albens* | 12 | 0 | Perennial |  |  |
| *Dianthus caespitosus* | 12 | 0 | Perennial |  |  |
| *Dianthus crenatus* | 12 | 0 | Perennial |  |  |
| *Silene atlantica* | 12 | 0 | Perennial |  |  |
| *Silene bellidioides* | 12 | 0 | Perennial |  |  |
| *Silene caesia* | 12 | 0 | Perennial |  |  |
| *Silene cinerea* | 12 | 0 | Perennial |  |  |
| *Silene densifolia* | 12 | 0 | Perennial |  |  |
| *Silene filifolia* | 12 | 0 | Perennial |  |  |
| *Silene heldreichii* | 12 | 0 | Annual |  |  |
| *Silene iberica* | 12 | 0 | Annual |  |  |
| *Silene lithophila* | 12 | 0 | Perennial |  |  |
| *Silene livida* | 12 | 0 | Perennial |  |  |
| *Silene pentelica* | 12 | 0 | Annual |  |  |
| *Silene phoenicodonta* | 12 | 0 | Perennial | 3 | 5 |
| *Silene thymifolia* | 12 | 0 | Perennial |  |  |
| *Petrorhagia saxifraga* | 11 | 0 | Perennial |  |  |
| *Silene ammophila* | 11 | 0 | Annual |  |  |
| *Silene attenuata* | 11 | 1 | Perennial |  |  |
| *Silene burmanica* | 11 | 0 | Perennial |  |  |
| *Silene choulettii* | 11 | 0 | Perennial |  |  |
| *Silene lomalasinense* | 11 | 0 | Perennial |  |  |
| *Silene martyii* | 11 | 0 | Perennial |  |  |
| *Silene pharnaceifolia* | 11 | 1 | Perennial |  |  |
| *Silene reichenbrachii* | 11 | 0 | Perennial |  |  |
| *Silene skorpili* | 11 | 0 | Perennial |  |  |
| *Silene wahlbergella* | 11 | 0 | Perennial |  |  |
| *Calandrinia arenaria* | 10 | 0 | Annual |  |  |
| *Calandrinia breweri* | 10 | 0 | Annual |  |  |
| *Calandrinia picta* | 10 | 0 | Perennial |  |  |
| *Dianthus broteri* | 10 | 0 | Perennial |  |  |
| *Dianthus carthusianorum* | 10 | 0 | Perennial |  |  |
| *Dianthus deltoides* | 10 | 0 | Perennial |  |  |
| *Dianthus scaber* | 10 | 0 | Perennial |  |  |
| *Lychnis stellarioides* | 10 | 1 | Perennial |  |  |
| *Petrorhagia prolifera* | 10 | 0 | Annual |  |  |
| *Silene caspica* | 10 | 0 | Perennial |  |  |
| *Silene fissipetala* | 10 | 0 | Perennial |  |  |
| *Silene friwaldszkyana* | 10 | 0 | Perennial |  |  |
| *Silene gavrilovii* | 10 | 0 | Perennial |  |  |
| *Silene holopetala* | 10 | 0 | Perennial | 3 |  |
| *Silene latifolia/dioica* | 10 | 0 | Perennial |  |  |
| *Silene morrisonmontana* | 10 | 0 | Perennial | 1 | 5 |
| *Silene nachlingerae* | 10 | 0 | Perennial |  |  |
| *Silene oropediorum* | 10 | 0 | Annual |  |  |
| *Silene palaestina* | 10 | 0 | Annual |  |  |
| *Silene requienii* | 10 | 0 | Perennial |  |  |
| *Silene tolmatchevii* | 10 | 0 | Perennial |  |  |
| *Silene trachyphylla* | 10 | 0 | Perennial | 4 | 9 |
| *Silene variegata* | 10 | 0 | Perennial |  |  |
| *Silene yetii* | 10 | 0 | Perennial | 4 |  |
| *Calandrinia ambigua* | 9 | 0 | Annual |  |  |
| *Calandrinia axilliflora* | 9 | 0 | Annual |  |  |
| *Calandrinia calycina* | 9 | 0 | Annual |  |  |
| *Silene ajanensis* | 9 | 0 | Perennial |  |  |
| *Silene argaea* | 9 | 1 | Perennial | 3 |  |
| *Silene chirensis* | 9 | 0 | Perennial |  |  |
| *Silene filipetala* | 9 | 0 | Perennial |  |  |
| *Silene gebleriana* | 9 | 0 | Perennial |  |  |
| *Silene grisea* | 9 | 0 | Perennial |  |  |
| *Silene hawaiiensis* | 9 | 0 | Perennial |  |  |
| *Silene invisa* | 9 | 0 | Perennial | 2 |  |
| *Silene lacera* | 9 | 1 | Perennial | 1 |  |
| *Silene nivalis* | 9 | 0 | Perennial |  |  |
| *Silene olgae* | 9 | 0 | Perennial |  |  |
| *Silene rechingeri* | 9 | 0 | Perennial |  |  |
| *Silene vautierae* | 9 | 0 | Perennial |  |  |
| *Calandrinia celosioides* | 8 | 0 | Perennial |  |  |
| *Silene arenarioides* | 8 | 0 | Perennial |  |  |
| *Silene auriculata* | 8 | 0 | Perennial |  |  |
| *Silene baschkirorum* | 8 | 0 | Perennial |  |  |
| *Silene caesarea* | 8 | 0 | Perennial |  |  |
| *Silene caucasica* | 8 | 0 | Perennial |  |  |
| *Silene cyri* | 8 | 0 | Perennial |  |  |
| *Silene favargeri* | 8 | 2 | Perennial |  |  |
| *Silene guntensis* | 8 | 0 | Perennial |  |  |
| *Silene lazica* | 8 | 0 | Perennial |  |  |
| *Silene ovalifolia* | 8 | 0 | Perennial |  |  |
| *Silene parnassica* | 8 | 1 | Perennial | 3 |  |
| *Silene pinetorum* | 8 | 0 | Annual |  |  |
| *Silene pseudotites* | 8 | 0 | Perennial |  |  |
| *Silene pygmaea* | 8 | 0 | Perennial |  |  |
| *Silene sennenii* | 8 | 0 | Perennial | 1 | 8 |
| *Silene sieberi* | 8 | 0 | Perennial |  |  |
| *Silene tagadirtensis* | 8 | 0 | Perennial |  |  |
| *Silene turgida* | 8 | 0 | Perennial |  |  |
| *Silene wrightii* | 8 | 1 | Perennial | 2 | 7 |
| *Viscaria asterias* | 8 | 0 | Perennial |  |  |
| *Calandrinia lingulata* | 7 | 0 | Unclassified |  |  |
| *Ceraria namaquensis* | 7 | 0 | Perennial |  |  |
| *Dianthus micropetalus* | 7 | 0 | Perennial |  |  |
| *Saponaria glutinosa* | 7 | 0 | Annual |  |  |
| *Saponaria orientalis* | 7 | 0 | Annual |  |  |
| *Silene brevicaulis* | 7 | 0 | Perennial |  |  |
| *Silene cancellata* | 7 | 0 | Perennial |  |  |
| *Silene cretacea* | 7 | 0 | Perennial |  |  |
| *Silene depressa* | 7 | 0 | Perennial |  |  |
| *Silene dubia* | 7 | 0 | Perennial |  |  |
| *Silene esquamata* | 7 | 0 | Perennial | 4 | 10 |
| *Silene fargesii* | 7 | 0 | Unclassified |  |  |
| *Silene fimbriata* | 7 | 0 | Perennial |  |  |
| *Silene glabrescens* | 7 | 0 | Annual |  |  |
| *Silene glareosa* | 7 | 0 | Perennial |  |  |
| *Silene gysophila* | 7 | 0 | Perennial |  |  |
| *Silene hupehensis* | 7 | 0 | Perennial | 4 | 8 |
| *Silene mekinensis* | 7 | 0 | Annual |  |  |
| *Silene niederi* | 7 | 0 | Perennial |  |  |
| *Silene oblanceolata* | 7 | 0 | Perennial | 4 | 10 |
| *Silene obtusidentata* | 7 | 0 | Perennial |  |  |
| *Silene pseudovelutina* | 7 | 0 | Perennial |  |  |
| *Silene requiemii* | 7 | 0 | Perennial |  |  |
| *Silene salzmannii* | 7 | 0 | Perennial |  |  |
| *Silene subcretacea* | 7 | 0 | Annual |  |  |
| *Silene suffructescens* | 7 | 0 | Perennial |  |  |
| *Silene thunbergiana* | 7 | 0 | Perennial |  |  |
| *Silene virescens* | 7 | 0 | Annual |  |  |
| *Agrostemma githago* | 6 | 0 | Annual |  |  |
| *Calandrinia amarantoides* | 6 | 0 | Unclassified |  |  |
| *Calandrinia coquimbensis* | 6 | 0 | Annual |  |  |
| *Calandrinia eremaea* | 6 | 0 | Annual |  |  |
| *Ceraria carrissoana* | 6 | 0 | Perennial |  |  |
| *Dianthus arenarius* | 6 | 0 | Perennial |  |  |
| *Dianthus armeria* | 6 | 0 | Annual |  |  |
| *Lychnis grandifolia* | 6 | 0 | Perennial |  |  |
| *Lychnis haagena* | 6 | 0 | Perennial |  |  |
| *Lychnis wilfordii* | 6 | 0 | Perennial | 4 | 13 |
| *Saponaria calabrica* | 6 | 0 | Annual |  |  |
| *Silene adscendens* | 6 | 0 | Annual |  |  |
| *Silene argentina* | 6 | 0 | Perennial |  |  |
| *Silene atrocastanea* | 6 | 0 | Perennial | 3 | 8 |
| *Silene ayachica* | 6 | 0 | Perennial |  |  |
| *Silene brachypetala* | 6 | 0 | Annual |  |  |
| *Silene cattariniana* | 6 | 0 | Perennial |  |  |
| *Silene cisplatensis* | 6 | 0 | Annual |  |  |
| *Silene colpophylla* | 6 | 0 | Perennial |  |  |
| *Silene echinata* | 6 | 0 | Annual |  |  |
| *Silene eduardii* | 6 | 0 | Perennial |  |  |
| *Silene genistifolia* | 6 | 0 | Perennial |  |  |
| *Silene gibraltarica* | 6 | 0 | Perennial |  |  |
| *Silene hieranymi* | 6 | 0 | Perennial |  |  |
| *Silene keiskei* | 6 | 0 | Perennial |  |  |
| *Silene macrorhiza* | 6 | 0 | Perennial |  |  |
| *Silene mariana* | 6 | 0 | Annual |  |  |
| *Silene mentagensis* | 6 | 0 | Perennial |  |  |
| *Silene microphylla* | 6 | 0 | Perennial |  |  |
| *Silene monbeigii* | 6 | 0 | Perennial | 4 | 6 |
| *Silene nevadensis* | 6 | 0 | Perennial |  |  |
| *Silene plankii* | 6 | 0 | Perennial | 4 | 9 |
| *Silene praemixta* | 6 | 0 | Perennial |  |  |
| *Silene solenantha* | 6 | 1 | Perennial |  |  |
| *Silene stewartiana* | 6 | 0 | Perennial | 3 | 10 |
| *Calandrinia calyptrata* | 5 | 0 | Annual |  |  |
| *Dianthus bicolor* | 5 | 0 | Unclassified |  |  |
| *Lychnis lagragei* | 5 | 0 | Perennial |  |  |
| *Saponaria budilifolia* | 5 | 0 | Unclassified |  |  |
| *Silene articulata* | 5 | 0 | Perennial |  |  |
| *Silene barrattei* | 5 | 0 | Perennial |  |  |
| *Silene canariensis* | 5 | 0 | Perennial |  |  |
| *Silene capitata* | 5 | 0 | Perennial | 3 |  |
| *Silene cerastoides* | 5 | 0 | Annual |  |  |
| *Silene fabarioides* | 5 | 0 | Perennial |  |  |
| *Silene gangotriana* | 5 | 0 | Perennial |  |  |
| *Silene gillettii* | 5 | 0 | Perennial |  |  |
| *Silene khasiana* | 5 | 0 | Perennial | 4 |  |
| *Silene kuschakewiczii* | 5 | 1 | Perennial |  |  |
| *Silene lanceolata* | 5 | 0 | Perennial |  |  |
| *Silene lichiangensis* | 5 | 0 | Perennial | 4 | 6 |
| *Silene namlaensis* | 5 | 0 | Perennial | 3 |  |
| *Silene oreophila* | 5 | 0 | Perennial |  |  |
| *Silene phrygia* | 5 | 0 | Perennial |  |  |
| *Silene pompeiopolitana* | 5 | 0 | Annual |  |  |
| *Silene principis* | 5 | 1 | Perennial | 2 | 7 |
| *Silene rubricalyx* | 5 | 0 | Perennial | 4 | 5 |
| *Silene ruinarum* | 5 | 0 | Perennial |  |  |
| *Silene setisperma* | 5 | 0 | Perennial |  |  |
| *Silene sisianica* | 5 | 0 | Perennial |  |  |
| *Silene tejedensis* | 5 | 0 | Perennial |  |  |
| *Silene trajectorum* | 5 | 0 | Perennial |  |  |
| *Calandrinia balonensis* | 4 | 0 | Annual |  |  |
| *Calandrinia longiscapa* | 4 | 0 | Annual |  |  |
| *Calandrinia monosperma* | 4 | 0 | Annual |  |  |
| *Calandrinia umbellata* | 4 | 0 | Perennial |  |  |
| *Ceraria longipedunculata* | 4 | 0 | Perennial |  |  |
| *Cistanthe ambigua* | 4 | 0 | Annual |  |  |
| *Dianthus ciliatus* | 4 | 0 | Unclassified |  |  |
| *Dianthus crinitus* | 4 | 0 | Perennial |  |  |
| *Dianthus laingsburgensis* | 4 | 0 | Perennial |  |  |
| *Dianthus laricifolius* | 4 | 0 | Unclassified |  |  |
| *Dianthus thunbergii* | 4 | 0 | Perennial |  |  |
| *Dianthus virgineus* | 4 | 0 | Perennial |  |  |
| *Saponaria chloraefolia* | 4 | 0 | Annual |  |  |
| *Saponaria mesogitana* | 4 | 0 | Annual |  |  |
| *Saponaria pulvinaris* | 4 | 0 | Perennial |  |  |
| *Saponaria vaccaria* | 4 | 0 | Annual |  |  |
| *Silene abietum* | 4 | 0 | Perennial |  |  |
| *Silene angustiflorum* | 4 | 0 | Perennial |  |  |
| *Silene bourgaei* | 4 | 0 | Perennial |  |  |
| *Silene cephalantha* | 4 | 1 | Perennial |  |  |
| *Silene citrina* | 4 | 0 | Perennial |  |  |
| *Silene eckloniana* | 4 | 0 | Perennial |  |  |
| *Silene elegans* | 4 | 0 | Perennial |  |  |
| *Silene goulimyi* | 4 | 0 | Perennial |  |  |
| *Silene hochstetteri* | 4 | 0 | Perennial |  |  |
| *Silene hoefftiana* | 4 | 0 | Perennial |  |  |
| *Silene holzmannii* | 4 | 0 | Annual |  |  |
| *Silene laxa* | 4 | 0 | Perennial |  |  |
| *Silene linoides* | 4 | 0 | Perennial |  |  |
| *Silene litwinovii* | 4 | 0 | Perennial |  |  |
| *Silene lychnidea* | 4 | 0 | Perennial |  |  |
| *Silene orphamidis* | 4 | 0 | Perennial |  |  |
| *Silene oxyodonta* | 4 | 0 | Annual |  |  |
| *Silene patagonica* | 4 | 0 | Perennial |  |  |
| *Silene pilosellaefolia* | 4 | 0 | Perennial |  |  |
| *Silene pseudobehen* | 4 | 0 | Annual |  |  |
| *Silene scabrida* | 4 | 0 | Annual |  |  |
| *Silene schwarzerbegeri* | 4 | 0 | Perennial |  |  |
| *Silene splendens* | 4 | 0 | Perennial |  |  |
| *Silene stenocalycina* | 4 | 0 | Perennial |  |  |
| *Silene striata* | 4 | 0 | Annual |  |  |
| *Silene tragacantha* | 4 | 0 | Perennial |  |  |
| *Silene tunetana* | 4 | 0 | Annual |  |  |
| *Silene tunicoides* | 4 | 0 | Perennial |  |  |
| *Silene wardii* | 4 | 0 | Perennial | 3 |  |
| *Silene yarmalii* | 4 | 0 | Unclassified |  |  |
| *Silene yemensis* | 4 | 0 | Perennial |  |  |
| *Calandrinia cymosa* | 3 | 0 | Annual |  |  |
| *Calandrinia liniflora* | 3 | 0 | Annual |  |  |
| *Calandrinia littoralis* | 3 | 0 | Annual |  |  |
| *Calandrinia ptychosperma* | 3 | 0 | Annual |  |  |
| *Calandrinia rosea* | 3 | 0 | Annual |  |  |
| *Calandrinia uniflora* | 3 | 0 | Annual |  |  |
| *Calandrinia weberensis* | 3 | 0 | Unclassified |  |  |
| *Ceraria fruticulosa* | 3 | 0 | Perennial |  |  |
| *Dianthus actinopetalus* | 3 | 0 | Unclassified |  |  |
| *Dianthus anatolicus* | 3 | 0 | Unclassified |  |  |
| *Dianthus angolensis* | 3 | 0 | Perennial |  |  |
| *Dianthus bolusii* | 3 | 0 | Perennial |  |  |
| *Dianthus diminitus* | 3 | 0 | Unclassified |  |  |
| *Dianthus excelsus* | 3 | 0 | Perennial |  |  |
| *Dianthus longicaulis* | 3 | 0 | Perennial |  |  |
| *Lychnis fulgens x coronata* | 3 | 0 | Perennial |  |  |
| *Lychnis montana* | 3 | 0 | Perennial |  |  |
| *Saponaria prostrata* | 3 | 0 | Unclassified |  |  |
| *Silene alexandri* | 3 | 0 | Perennial |  |  |
| *Silene anisoloba* | 3 | 0 | Perennial |  |  |
| *Silene argentinensis* | 3 | 0 | Perennial |  |  |
| *Silene aristides* | 3 | 0 | Perennial |  |  |
| *Silene balcanica* | 3 | 0 | Perennial |  |  |
| *Silene berlandier* | 3 | 0 | Unclassified |  |  |
| *Silene brotherana* | 3 | 0 | Perennial |  |  |
| *Silene chersonensis* | 3 | 0 | Perennial |  |  |
| *Silene chubutensis* | 3 | 0 | Perennial |  |  |
| *Silene claviformis* | 3 | 0 | Perennial |  |  |
| *Silene crassicaulis* | 3 | 0 | Perennial |  |  |
| *Silene cryptantha* | 3 | 0 | Perennial |  |  |
| *Silene eremitica* | 3 | 0 | Perennial |  |  |
| *Silene euxina* | 3 | 0 | Perennial |  |  |
| *Silene exaltata* | 3 | 0 | Annual |  |  |
| *Silene excedens* | 3 | 0 | Perennial |  |  |
| *Silene fongesii* | 3 | 0 | Unclassified |  |  |
| *Silene gaditana* | 3 | 0 | Annual |  |  |
| *Silene gertraudiae* | 3 | 0 | Perennial |  |  |
| *Silene hifacensis* | 3 | 0 | Perennial | 3 | 8 |
| *Silene huguettiae* | 3 | 0 | Perennial | 2 |  |
| *Silene humilus* | 3 | 0 | Perennial |  |  |
| *Silene hussoni* | 3 | 0 | Annual |  |  |
| *Silene insularis* | 3 | 0 | Annual |  |  |
| *Silene koreana* | 3 | 0 | Annual |  |  |
| *Silene leptoclada* | 3 | 0 | Perennial |  |  |
| *Silene leptopetala* | 3 | 0 | Perennial |  |  |
| *Silene leucophylla* | 3 | 0 | Perennial |  |  |
| *Silene lhassana* | 3 | 0 | Perennial | 3 | 5 |
| *Silene manissadjiana* | 3 | 0 | Perennial |  |  |
| *Silene mesatlantica* | 3 | 0 | Perennial |  |  |
| *Silene nocteolens* | 3 | 0 | Perennial |  |  |
| *Silene notarisii* | 3 | 0 | Perennial | 1 | 7 |
| *Silene palinotricha* | 3 | 0 | Perennial |  |  |
| *Silene propinqua* | 3 | 0 | Perennial |  |  |
| *Silene pseudonutans* | 3 | 0 | Perennial |  |  |
| *Silene remotiflora* | 3 | 0 | Annual |  |  |
| *Silene rosiflora* | 3 | 0 | Perennial | 4 | 5 |
| *Silene rouyana* | 3 | 0 | Perennial |  |  |
| *Silene sarawschanica* | 3 | 1 | Perennial |  | 5 |
| *Silene scabrifolia* | 3 | 0 | Perennial |  |  |
| *Silene sibthorpiana* | 3 | 0 | Annual |  |  |
| *Silene tianschanica* | 3 | 0 | Perennial | 1 |  |
| *Silene tomentosa* | 3 | 0 | Perennial | 3 | 8 |
| *Silene triflora* | 3 | 0 | Perennial |  |  |
| *Silene uncerntae* | 3 | 0 | Unclassified |  |  |
| *Silene watsonii* | 3 | 0 | Perennial |  |  |
| *Calandrinia cephalophora* | 2 | 0 | Unclassified |  |  |
| *Calandrinia chrysantha* | 2 | 0 | Perennial |  |  |
| *Calandrinia densiflora* | 2 | 0 | Annual |  |  |
| *Calandrinia denticulata* | 2 | 0 | Perennial |  |  |
| *Calandrinia fenzlii* | 2 | 0 | Perennial |  |  |
| *Calandrinia parryi* | 2 | 0 | Annual |  |  |
| *Calandrinia pulchella* | 2 | 0 | Annual |  |  |
| *Calandrinia pumila* | 2 | 0 | Annual |  |  |
| *Calandrinia quadripetalum* | 2 | 0 | Annual |  |  |
| *Calandrinia tweedyi* | 2 | 0 | Perennial |  |  |
| *Calyptrotheca taitensis* | 2 | 0 | Perennial |  |  |
| *Dianthus abyssinicus* | 2 | 0 | Unclassified |  |  |
| *Dianthus acicularis* | 2 | 0 | Perennial |  |  |
| *Dianthus attenuatus* | 2 | 0 | Perennial |  |  |
| *Dianthus borbassi* | 2 | 0 | Unclassified |  |  |
| *Dianthus gaditanus* | 2 | 0 | Perennial |  |  |
| *Dianthus kamisbegensis* | 2 | 0 | Unclassified |  |  |
| *Dianthus multiceps* | 2 | 0 | Unclassified |  |  |
| *Dianthus polymorphus* | 2 | 0 | Perennial |  |  |
| *Dianthus prostratus* | 2 | 0 | Perennial |  |  |
| *Dianthus siculus* | 2 | 0 | Perennial |  |  |
| *Dianthus transvaalensis* | 2 | 0 | Perennial |  |  |
| *Lychnis abyssinica* | 2 | 0 | Perennial |  |  |
| *Lychnis kiwuensis* | 2 | 0 | Perennial |  |  |
| *Lychnis sieboldii* | 2 | 0 | Perennial |  |  |
| *Lychnis sordida* | 2 | 0 | Perennial |  |  |
| *Saponaria cerastioides* | 2 | 0 | Unclassified |  |  |
| *Saponaria graeca* | 2 | 0 | Annual |  |  |
| *Saponaria kotschyi* | 2 | 0 | Annual |  |  |
| *Silene acratha* | 2 | 0 | Unclassified |  |  |
| *Silene adenocalyx* | 2 | 0 | Perennial | 3 | 5 |
| *Silene alaschanica* | 2 | 0 | Perennial | 3 |  |
| *Silene andicola* | 2 | 0 | Perennial |  |  |
| *Silene antarctica* | 2 | 0 | Perennial |  |  |
| *Silene atsaensis* | 2 | 0 | Perennial | 2 | 7 |
| *Silene baccifera* | 2 | 0 | Perennial | 1 | 10 |
| *Silene bersieri* | 2 | 0 | Perennial |  |  |
| *Silene bilingua* | 2 | 0 | Perennial | 4 | 4 |
| *Silene bosniaca* | 2 | 0 | Perennial |  |  |
| *Silene breviauriculata* | 2 | 0 | Perennial |  |  |
| *Silene capitellata* | 2 | 0 | Perennial |  |  |
| *Silene cephallenia* | 2 | 0 | Perennial |  |  |
| *Silene chamarensis* | 2 | 0 | Perennial |  |  |
| *Silene chungtienensis* | 2 | 0 | Perennial | 3 | 4 |
| *Silene cobalticola* | 2 | 0 | Perennial |  |  |
| *Silene corymbosa* | 2 | 0 | Unclassified |  |  |
| *Silene crispans* | 2 | 0 | Perennial |  |  |
| *Silene depauperata* | 2 | 0 | Unclassified |  |  |
| *Silene dschuparensis* | 2 | 0 | Perennial |  |  |
| *Silene echinus* | 2 | 0 | Perennial |  |  |
| *Silene erysimifolia* | 2 | 0 | Perennial |  |  |
| *Silene fernandezii* | 2 | 0 | Perennial | 2 | 9 |
| *Silene fetissovii* | 2 | 0 | Perennial |  |  |
| *Silene giraldii* | 2 | 0 | Annual |  |  |
| *Silene glutinosa* | 2 | 0 | Perennial |  |  |
| *Silene gomeraea* | 2 | 0 | Unclassified |  |  |
| *Silene goniocaula* | 2 | 0 | Perennial |  |  |
| *Silene hayekiana* | 2 | 0 | Perennial |  |  |
| *Silene hellmannii* | 2 | 0 | Perennial |  |  |
| *Silene incentae* | 2 | 0 | Unclassified |  |  |
| *Silene indeprensa* | 2 | 0 | Perennial |  |  |
| *Silene ionica* | 2 | 0 | Perennial |  |  |
| *Silene iranica* | 2 | 0 | Unclassified |  |  |
| *Silene jugorum* | 2 | 0 | Perennial |  |  |
| *Silene kialensis* | 2 | 0 | Perennial | 3 | 4 |
| *Silene kumaonensis* | 2 | 0 | Perennial |  |  |
| *Silene lasiostyla* | 2 | 0 | Annual |  |  |
| *Silene leptocaulis* | 2 | 0 | Perennial |  |  |
| *Silene maurisca* | 2 | 0 | Perennial |  |  |
| *Silene melandroides* | 2 | 0 | Perennial |  |  |
| *Silene oberacea* | 2 | 0 | Unclassified |  |  |
| *Silene oligantha* | 2 | 0 | Perennial |  |  |
| *Silene ordossica* | 2 | 0 | Perennial |  |  |
| *Silene ouensae* | 2 | 0 | Perennial |  |  |
| *Silene patula X rosulata* | 2 | 0 | Perennial |  |  |
| *Silene pseudofortunei* | 2 | 0 | Perennial | 3 |  |
| *Silene quadriloba* | 2 | 0 | Annual |  |  |
| *Silene reinwardtii* | 2 | 0 | Annual |  |  |
| *Silene riphaena* | 2 | 0 | Unclassified |  |  |
| *Silene roopiana* | 2 | 0 | Perennial |  |  |
| *Silene salicifolia* | 2 | 0 | Perennial | 3 | 7 |
| *Silene sangaria* | 2 | 0 | Perennial |  |  |
| *Silene schimperiana* | 2 | 0 | Perennial |  |  |
| *Silene semenovii* | 2 | 0 | Perennial |  |  |
| *Silene seoulensis* | 2 | 0 | Perennial | 1 |  |
| *Silene siderophila* | 2 | 0 | Perennial |  |  |
| *Silene sussamyrica* | 2 | 0 | Perennial |  |  |
| *Silene terroveana* | 2 | 0 | Unclassified |  |  |
| *Silene thirkeana* | 2 | 0 | Annual |  |  |
| *Silene thomsonii* | 2 | 0 | Perennial |  |  |
| *Silene turkestanica* | 2 | 0 | Perennial |  |  |
| *Silene undulatifolia* | 2 | 0 | Annual |  |  |
| *Silene velutinoides* | 2 | 0 | Perennial |  |  |
| *Silene vidaliana* | 2 | 0 | Perennial |  |  |
| *Atocion asterias* | 1 | 0 | Perennial |  |  |
| *Calandrinia composita* | 1 | 0 | Annual |  |  |
| *Calandrinia corrigioloides* | 1 | 0 | Annual |  |  |
| *Calandrinia ferruginea* | 1 | 0 | Perennial |  |  |
| *Calandrinia fuegrana* | 1 | 0 | Unclassified |  |  |
| *Calandrinia gilliesii* | 1 | 0 | Perennial |  |  |
| *Calandrinia glauca* | 1 | 0 | Perennial |  |  |
| *Calandrinia megarhiza* | 1 | 0 | Perennial |  |  |
| *Calandrinia minuscula* | 1 | 0 | Annual |  |  |
| *Calandrinia oblonga* | 1 | 0 | Unclassified |  |  |
| *Calandrinia oligantha* | 1 | 0 | Unclassified |  |  |
| *Calandrinia parviflora* | 1 | 0 | Unclassified |  |  |
| *Calandrinia patagonica* | 1 | 0 | Unclassified |  |  |
| *Calandrinia pickeringii* | 1 | 0 | Perennial |  |  |
| *Calandrinia pleiopetala* | 1 | 0 | Annual |  |  |
| *Calandrinia polia* | 1 | 0 | Perennial |  |  |
| *Calandrinia polyandra* | 1 | 0 | Annual |  |  |
| *Calandrinia polycarpoides* | 1 | 0 | Annual |  |  |
| *Calandrinia primuliflora* | 1 | 0 | Annual |  |  |
| *Calandrinia quadrivalvis* | 1 | 0 | Annual |  |  |
| *Calandrinia remota* | 1 | 0 | Annual |  |  |
| *Calandrinia reticulata* | 1 | 0 | Annual |  |  |
| *Calandrinia saltensis* | 1 | 0 | Unclassified |  |  |
| *Calandrinia spergularina* | 1 | 0 | Annual |  |  |
| *Calandrinia thyrsoides* | 1 | 0 | Annual |  |  |
| *Calandrinia volubilis* | 1 | 0 | Annual |  |  |
| *Calyptrotheca somalensis* | 1 | 0 | Perennial |  |  |
| *Dianthus acantholimonoides* | 1 | 0 | Unclassified |  |  |
| *Dianthus amurensis* | 1 | 0 | Perennial |  |  |
| *Dianthus andrzevowskianus* | 1 | 0 | Unclassified |  |  |
| *Dianthus angrenicus* | 1 | 0 | Unclassified |  |  |
| *Dianthus asperulus* | 1 | 0 | Unclassified |  |  |
| *Dianthus atomarius* | 1 | 0 | Unclassified |  |  |
| *Dianthus baldhuanicus* | 1 | 0 | Unclassified |  |  |
| *Dianthus brevicaulis* | 1 | 0 | Unclassified |  |  |
| *Dianthus coeli-rosa* | 1 | 0 | Unclassified |  |  |
| *Dianthus crassipes* | 1 | 0 | Unclassified |  |  |
| *Dianthus cyri* | 1 | 0 | Annual |  |  |
| *Dianthus gallicus* | 1 | 0 | Perennial |  |  |
| *Dianthus holopetalus* | 1 | 0 | Perennial |  |  |
| *Dianthus leptoloma* | 1 | 0 | Unclassified |  |  |
| *Dianthus liburnicus* | 1 | 0 | Perennial |  |  |
| *Dianthus longiglumis* | 1 | 0 | Perennial |  |  |
| *Dianthus macalisberg* | 1 | 0 | Unclassified |  |  |
| *Dianthus macropetalus* | 1 | 0 | Unclassified |  |  |
| *Dianthus niculus* | 1 | 0 | Unclassified |  |  |
| *Dianthus plumarius* | 1 | 0 | Perennial |  |  |
| *Dianthus pseudo-arineria* | 1 | 0 | Unclassified |  |  |
| *Dianthus rigidus* | 1 | 0 | Perennial |  |  |
| *Dianthus setabensis* | 1 | 0 | Unclassified |  |  |
| *Dianthus subimbricalus* | 1 | 0 | Unclassified |  |  |
| *Dianthus velutinus* | 1 | 0 | Annual |  |  |
| *Heliosperma chromodontum* | 1 | 0 | Perennial |  |  |
| *Heliosperma pudibunda* | 1 | 0 | Perennial |  |  |
| *Heliosperma veselskyi* | 1 | 0 | Perennial |  |  |
| *Lychnis chiranthiflora* | 1 | 0 | Annual |  |  |
| *Lychnis dawsonii* | 1 | 0 | Perennial |  |  |
| *Lychnis gillettii* | 1 | 0 | Perennial |  |  |
| *Lychnis japonica* | 1 | 0 | Perennial |  |  |
| *Lychnis kiusiana* | 1 | 0 | Perennial |  |  |
| *Lychnis longata* | 1 | 0 | Unclassified |  |  |
| *Lychnis praecox* | 1 | 0 | Perennial |  |  |
| *Lychnis preslii* | 1 | 0 | Perennial |  |  |
| *Petrocoptis glaucifolia* | 1 | 0 | Perennial |  |  |
| *Petrocoptis glaucifolia* | 1 | 0 | Perennial |  |  |
| *Saponaria acuminoides* | 1 | 0 | Unclassified |  |  |
| *Saponaria alpina* | 1 | 0 | Unclassified |  |  |
| *Saponaria ammophila* | 1 | 0 | Unclassified |  |  |
| *Saponaria bodeana* | 1 | 0 | Unclassified |  |  |
| *Saponaria cypria* | 1 | 0 | Unclassified |  |  |
| *Saponaria depressa* | 1 | 0 | Perennial |  |  |
| *Silene adusta* | 1 | 0 | Annual |  |  |
| *Silene aequipetala* | 1 | 0 | Unclassified |  |  |
| *Silene affghanica* | 1 | 0 | Perennial |  |  |
| *Silene alexandrae* | 1 | 0 | Annual |  |  |
| *Silene amata* | 1 | 0 | Unclassified |  |  |
| *Silene araratica* | 1 | 0 | Perennial |  |  |
| *Silene arenaica* | 1 | 0 | Perennial |  |  |
| *Silene arghurica* | 1 | 0 | Unclassified |  |  |
| *Silene assyriaca* | 1 | 0 | Annual |  |  |
| *Silene atropatana* | 1 | 0 | Unclassified |  |  |
| *Silene austro-iranica* | 1 | 0 | Annual |  |  |
| *Silene balansae* | 1 | 0 | Perennial |  |  |
| *Silene barbata* | 1 | 0 | Unclassified |  |  |
| *Silene bassanensis* | 1 | 0 | Perennial |  |  |
| *Silene berthelotiana* | 1 | 0 | Perennial |  |  |
| *Silene bodinieri* | 1 | 0 | Perennial |  |  |
| *Silene brevicalyx* | 1 | 0 | Perennial |  |  |
| *Silene brevipes* | 1 | 0 | Unclassified |  |  |
| *Silene brigittae* | 1 | 0 | Perennial |  |  |
| *Silene campreta* | 1 | 0 | Unclassified |  |  |
| *Silene cenarearia* | 1 | 0 | Unclassified |  |  |
| *Silene cernua* | 1 | 0 | Perennial |  |  |
| *Silene cheiranthifolia* | 1 | 0 | Annual |  |  |
| *Silene chihuahuensis* | 1 | 0 | Perennial |  |  |
| *Silene clokeyi* | 1 | 0 | Perennial |  |  |
| *Silene codonocalyx* | 1 | 0 | Unclassified |  |  |
| *Silene condonocalyx* | 1 | 0 | Unclassified |  |  |
| *Silene conspersa* | 1 | 0 | Unclassified |  |  |
| *Silene cornuta* | 1 | 0 | Unclassified |  |  |
| *Silene crerimutata* | 1 | 0 | Unclassified |  |  |
| *Silene cyrenaica* | 1 | 0 | Annual |  |  |
| *Silene dinarica* | 1 | 0 | Perennial |  |  |
| *Silene dinteri* | 1 | 0 | Perennial |  |  |
| *Silene divergens* | 1 | 0 | Unclassified |  |  |
| *Silene dyris* | 1 | 0 | Perennial |  |  |
| *Silene engleri* | 1 | 0 | Annual |  |  |
| *Silene erecta* | 1 | 0 | Unclassified |  |  |
| *Silene ereuricana* | 1 | 0 | Unclassified |  |  |
| *Silene eruscipula* | 1 | 0 | Annual |  |  |
| *Silene eviscosa* | 1 | 0 | Perennial |  |  |
| *Silene falconeri* | 1 | 0 | Perennial |  |  |
| *Silene falconerii* | 1 | 0 | Perennial |  |  |
| *Silene fissinervia* | 1 | 0 | Unclassified |  |  |
| *Silene flaccida* | 1 | 0 | Perennial |  |  |
| *Silene flexuosa* | 1 | 0 | Unclassified |  |  |
| *Silene formosa* | 1 | 0 | Unclassified |  |  |
| *Silene gaillardotiana* | 1 | 0 | Unclassified |  |  |
| *Silene galataea* | 1 | 0 | Perennial |  |  |
| *Silene gaubae* | 1 | 0 | Unclassified |  |  |
| *Silene gmelini* | 1 | 0 | Unclassified |  |  |
| *Silene graniticola* | 1 | 0 | Perennial |  |  |
| *Silene haesarensis* | 1 | 0 | Unclassified |  |  |
| *Silene haussknechtii* | 1 | 0 | Annual |  |  |
| *Silene helleboriflora* | 1 | 0 | Perennial |  |  |
| *Silene heterotricha* | 1 | 0 | Unclassified |  |  |
| *Silene hispanica* | 1 | 0 | Annual |  |  |
| *Silene hohenackeri* | 1 | 0 | Perennial |  |  |
| *Silene holosteifolia* | 1 | 0 | Perennial |  |  |
| *Silene humifusa* | 1 | 0 | Unclassified |  |  |
| *Silene hybrida* | 1 | 0 | Unclassified |  |  |
| *Silene ignobilis* | 1 | 0 | Annual |  |  |
| *Silene intricata* | 1 | 0 | Annual |  |  |
| *Silene intrusa* | 1 | 0 | Annual |  |  |
| *Silene ischnopetala* | 1 | 0 | Perennial |  |  |
| *Silene jamesii* | 1 | 0 | Unclassified |  |  |
| *Silene japonica* | 1 | 0 | Perennial |  |  |
| *Silene joerstadii* | 1 | 0 | Perennial |  |  |
| *Silene julina* | 1 | 0 | Unclassified |  |  |
| *Silene kuhistanica* | 1 | 0 | Perennial |  |  |
| *Silene lasiantha* | 1 | 0 | Perennial |  |  |
| *Silene leptophylla* | 1 | 0 | Unclassified |  |  |
| *Silene lermoria* | 1 | 0 | Unclassified |  |  |
| *Silene leucorosoa* | 1 | 0 | Unclassified |  |  |
| *Silene linae* | 1 | 0 | Perennial |  |  |
| *Silene lineata* | 1 | 0 | Perennial |  |  |
| *Silene littoralis* | 1 | 0 | Unclassified |  |  |
| *Silene longicalycina* | 1 | 0 | Perennial |  |  |
| *Silene longicarpophora* | 1 | 0 | Perennial |  |  |
| *Silene longisepala* | 1 | 0 | Perennial |  |  |
| *Silene luciliae* | 1 | 0 | Perennial |  |  |
| *Silene lupina* | 1 | 0 | Unclassified |  |  |
| *Silene lurida* | 1 | 0 | Perennial |  |  |
| *Silene lychnidiflora* | 1 | 0 | Annual |  |  |
| *Silene lyri* | 1 | 0 | Unclassified |  |  |
| *Silene macrosperma* | 1 | 0 | Perennial |  |  |
| *Silene madens* | 1 | 0 | Perennial |  |  |
| *Silene marcowiczii* | 1 | 0 | Perennial |  |  |
| *Silene margaritae* | 1 | 0 | Perennial |  |  |
| *Silene marmarica* | 1 | 0 | Perennial |  |  |
| *Silene masmenaea* | 1 | 0 | Perennial |  |  |
| *Silene maximowicziana* | 1 | 0 | Perennial |  |  |
| *Silene microloba* | 1 | 0 | Perennial |  |  |
| *Silene micropoda* | 1 | 0 | Unclassified |  |  |
| *Silene mongolica* | 1 | 0 | Perennial |  |  |
| *Silene morganae* | 1 | 0 | Perennial |  |  |
| *Silene mundiana* | 1 | 0 | Perennial |  |  |
| *Silene nangqenensis* | 1 | 0 | Perennial |  |  |
| *Silene natalii* | 1 | 0 | Unclassified |  |  |
| *Silene nebradensis* | 1 | 0 | Perennial |  |  |
| *Silene ningxiaensis* | 1 | 0 | Perennial | 1 |  |
| *Silene oretiva* | 1 | 0 | Unclassified |  |  |
| *Silene ostenfeldii* | 1 | 0 | Perennial | 2 | 3 |
| *Silene ovalis* | 1 | 0 | Unclassified |  |  |
| *Silene ovenda* | 1 | 0 | Unclassified |  |  |
| *Silene pakistanica* | 1 | 0 | Perennial |  |  |
| *Silene pamirensis* | 1 | 0 | Perennial |  |  |
| *Silene pariensis* | 1 | 0 | Unclassified |  |  |
| *Silene parjumanensis* | 1 | 0 | Perennial |  |  |
| *Silene patrini* | 1 | 0 | Unclassified |  |  |
| *Silene paucifolia* | 1 | 0 | Perennial |  |  |
| *Silene pedemontana* | 1 | 0 | Unclassified |  |  |
| *Silene pernoctans* | 1 | 0 | Unclassified |  |  |
| *Silene peton* | 1 | 0 | Unclassified |  |  |
| *Silene physocalyx* | 1 | 0 | Perennial |  |  |
| *Silene pilosella* | 1 | 0 | Unclassified |  |  |
| *Silene pseudotenuis* | 1 | 0 | Perennial | 2 |  |
| *Silene pseudoverticillata* | 1 | 0 | Perennial |  |  |
| *Silene psorophora* | 1 | 0 | Unclassified |  |  |
| *Silene pteroneura* | 1 | 0 | Perennial |  |  |
| *Silene reclinata* | 1 | 0 | Unclassified |  |  |
| *Silene refracta* | 1 | 0 | Unclassified |  |  |
| *Silene renzii* | 1 | 0 | Perennial |  |  |
| *Silene rubigena* | 1 | 0 | Perennial |  |  |
| *Silene ruthunica* | 1 | 0 | Perennial |  |  |
| *Silene sachalinensis* | 1 | 0 | Perennial |  |  |
| *Silene sanctae-therasiae* | 1 | 0 | Perennial |  |  |
| *Silene schmuckeri* | 1 | 0 | Perennial |  |  |
| *Silene schugnanica* | 1 | 0 | Perennial |  |  |
| *Silene sclerophylla* | 1 | 0 | Perennial |  |  |
| *Silene sicula* | 1 | 0 | Unclassified |  |  |
| *Silene singei* | 1 | 0 | Unclassified |  |  |
| *Silene soleracea* | 1 | 0 | Unclassified |  |  |
| *Silene spinosa* | 1 | 0 | Unclassified |  |  |
| *Silene stracheyi* | 1 | 0 | Perennial |  |  |
| *Silene sublanata* | 1 | 0 | Unclassified |  |  |
| *Silene tachtensis* | 1 | 0 | Perennial |  |  |
| *Silene thebana* | 1 | 0 | Perennial |  |  |
| *Silene tyrrhenia* | 1 | 0 | Perennial |  |  |
| *Silene ucrainica* | 1 | 0 | Perennial |  |  |
| *Silene vagans* | 1 | 0 | Perennial |  |  |
| *Silene valentina* | 1 | 0 | Unclassified |  |  |
| *Silene vesiculifera* | 1 | 0 | Annual |  |  |
| *Silene villoides* | 1 | 0 | Unclassified |  |  |
| *Silene vinosa* | 1 | 0 | Unclassified |  |  |
| *Silene virgata* | 1 | 0 | Annual |  |  |
| *Silene viscariopsis* | 1 | 0 | Perennial |  |  |
| *Silene vloki* | 1 | 0 | Perennial |  |  |
| *Silene waltoni* | 1 | 0 | Perennial |  |  |
| *Silene webbiana* | 1 | 0 | Unclassified |  |  |
