# Supplementary material for: Distribution of the anther-smut pathogen Microbotryum on species of the Caryophyllaceae
Source: New Phytol. 2010 Jul;187(1):217–29. doi: 10.1111/j.1469-8137.2010.03268.x (PMC3487183; doi:10.1111/j.1469-8137.2010.03268.x)
Supplement: Supplementary file 3 [file nph0187-0217-SD3.doc]

**Supporting Information Table S2**

| ***Sileneae* species GenBank NCBI accession numbers for DNA sequences of the *rps16* locus used for generating Fig. 1** | | | |
| --- | --- | --- | --- |
| Z83189.1 | *Silene acaulis* | AJ629915.1 | *Silene nigrescens* |
| EF061397.1 | *Silene portensis* | DQ908831.1 | *Silene nivea* |
| Z83158.1 | *Lychnis alpina* | Z83176.1 | *Silene noctiflora* |
| Z83193.2 | *Silene antirrhina* | Z83192.1 | *Silene nocturna* |
| Z83181.2 | *Silene aprica* | DQ908835.1 | *Silene oregana* |
| Z83159.1 | *Atocion armeria* | EF061393.1 | *Silene otites* |
| EF061360.1 | *Silene bupleuroides* | DQ908837.1 | *Silene parryi* |
| DQ908833.1 | *Silene nutans* | DQ908842.1 | *Silene repens* |
| DQ908814.1 | *Silene caroliniana* | DQ908841.1 | *Silene regia* |
| Z83156.1 | *Eudianthe coeli-rosa* | Z83173.1 | *Silene uniflora* |
| Z83170.1 | *Silene conica* | Z83160.1 | *Atocion rupestre* |
| DQ908818.1 | *Silene douglasii* | EF061394.1 | *Silene saxifraga* |
| DQ908819.1 | *Silene drummondii* | Z83179.1 | *Silene sedoides* |
| EF061385.1 | *Silene fortunei* | AJ831773.1 | *Silene sorensenis* |
| Z83154.1 | *Agrostemma githago* | DQ908847.1 | *Silene stellata* |
| EF061370.1 | *Silene inaperta* | AJ831765.1 | *Silene uralensis* |
| AJ831770.1 | *Silene involucrata* | DQ908852.1 | *Silene verecunda* |
| DQ908825.1 | *Silene laciniata* | AJ629912.1 | *Viscaria vulgaris* |
| Z83155.1 | *Eudianthe laeta* | DQ908854.1 | *Silene williamsii* |
| AY707940.1 | *Silene latifolia* | Z83172.1 | *Silene pendula* |
| Z83185.1 | *Silene littorea* | EF674192.1 | *Silene vulgaris* |
| Z83197.1 | *Silene muscipula* |  |  |
|  |  | | |
| ***Microbotryum* specimen GenBank NCBI accession numbers for DNA sequence of the ITS region used for generating Fig. 4** | | | |
| AY588083 | *Microbotryum* sp. from *Knautia arvensis* - Germany | | |
| GQ150527 | *Microbotryum* sp. form *Calandrinia affinis* herbarium specimen - Chile | | |
| GQ150529 | *Microbotryum* sp. form *Calandrinia colchanguensis* herbarium specimen - Chile | | |
| GQ150513 | *Microbotryum* sp. form *Dianthus neglectus* - near Vinadio, Italy | | |
| GQ150514 | *Microbotryum* sp. form *Dianthus sylvestris* - near La Grave, France | | |
| GQ150522 | *Microbotryum* sp. form *Lychnis cognata* herbarium specimen - China | | |
| GQ150526 | *Microbotryum* sp. form *Saponaria ocymoides* - Cesana Torinese, Italy | | |
| GQ150524 | *Microbotryum* sp. form *Silene acaulis* herbarium specimen - Alaska, USA | | |
| GQ150516 | *Microbotryum* sp. form *Silene acaulis* - Colorado, USA | | |
| GQ150515 | *Microbotryum* sp. form *Silene acaulis* - Monte Duca D'Abruzzi, Italy | | |
| GQ150518 | *Microbotryum* sp. form *Silene caroliniana* - Virginia Beach, USA | | |
| GQ150525 | *Microbotryum* sp. form *Silene chilensis* herbarium specimen - Chile | | |
| GQ150519 | *Microbotryum* sp. form *Silene dioica* - Bois Carre, France | | |
| GQ150517 | *Microbotryum* sp. form *Silene italica* herbarium specimen - France | | |
| GQ150520 | *Microbotryum* sp. form *Silene italica* - Santo Steffano, Italy | | |
| GQ150530 | *Microbotryum* sp. form *Silene lacera* herbarium specimen - Georgia | | |
| GQ150521 | *Microbotryum* sp. form *Silene latifolia* - Lamole, Italy | | |
| GQ150528 | *Microbotryum* sp. form *Silene paradoxa* - Lamole, Italy | | |
| GQ150523 | *Microbotryum* sp. form *Silene paryii* - Olympic Peninsula, USA | | |
